# ﻿Cryptic host-associated differentiation and diversity: unravelling the evolutionary dynamics of the plant pathogen *Lasiodiplodia*

**DOI:** 10.3897/imafungus.16.147543

**Published:** 2025-05-23

**Authors:** Ya-Zhu Ko, Huei-Chuan Shih, Meng-Shin Shiao, Yu-Chung Chiang

**Affiliations:** 1 Department of Biological Sciences, National Sun Yat-sen University, Kaohsiung 804, Taiwan National Sun Yat-sen University Kaohsiung Taiwan; 2 Department of Nursing, Meiho University, Pingtung 912, Taiwan Meiho University Pingtung Taiwan; 3 Research Laboratory Section, Offices of Health Science Research, Faculty of Medicine Ramathibodi Hospital, Mahidol University, Bangkok, Thailand Mahidol University Bangkok Thailand; 4 Department of Biomedical Science and Environment Biology, Kaohsiung Medical University, Kaohsiung 807, Taiwan Kaohsiung Medical University Kaohsiung Taiwan

**Keywords:** Fungal pathogens, genetic diversity, host-associated differentiation, *Lasiodiplodia*, microsatellite

## Abstract

*Lasiodiplodia*, a genus within the *Botryosphaeriaceae* family, comprises significant plant pathogens with a broad host range and global distribution, posing a substantial threat to agricultural production. Our recent study revealed the complexity of this genus by identifying numerous potential cryptic species within the seemingly generalist *L.theobromae*. To fully understand this species’ complexity, higher-resolution genetic markers are required. Therefore, this study employed a comprehensive analysis of multiple transferable microsatellite markers to verify *Lasiodiplodia* species delimitation and examine the fine-scale genetic structure and diversity of *Lasiodiplodia* species, particularly *L.theobromae*. The study identified four distinct genetic groups within *L.theobromae*, each showing high genetic diversity. The phylogenetic relationships of these groups align with the evolutionary history of their host plants. This finding suggests that host-pathogen co-evolution is shaped by shared ancestral variation, limited gene flow, isolation and natural selection. These insights enhance our understanding of managing economically important *Lasiodiplodia* plant pathogens and highlight the significance of genetic diversity and host preferences in developing effective control measures.

## ﻿Introduction

*Lasiodiplodia* encompasses numerous phytopathogenic fungal species causing various disease symptoms in different plant hosts in tropical and subtropical regions (Punithalingam 1980; Farr and Rossman 2021). The observed symptoms include fruits and seeds rot, necrotic lesions, neck rot, foliage yellowing, panicle brown rot, decline, branch dieback, stem-end rot, gummosis and stem canker (Burgess et al. 2006; Damm et al. 2007; Abdollahzadeh et al. 2010; de Oliveira Costa et al. 2010; Sakalidis et al. 2011; Ismail et al. 2012; Marques et al. 2013; Machado et al. 2014; Netto et al. 2014). Although *Lasiodiplodia* species were reported to be one of the most aggressive pathogens in *Botryosphaeriaceae*, various levels of aggressiveness were reported in different *Lasiodiplodia* species or isolates (de Oliveira Costa et al. 2010; Marques et al. 2013; Cruywagen et al. 2017; Rodríguez-Gálvez et al. 2017; Ali et al. 2020).

The classification of *Lasiodiplodia* species traditionally relies on several characteristics: the symptoms of infected plants, host pathogenicity, characteristics of pathogen culture and asexual reproduction types (Punithalingam 1976; Coutinho et al. 2017). However, these characteristics may vary under different environmental conditions. The variability complicates species identification and often leads to inconsistent conclusions (Coutinho et al. 2017). Recent studies have dedicated the effort to elucidate the phylogenetic relations of *Lasiodiplodia* species (Crous et al. 2006; Alves et al. 2008; Abdollahzadeh et al. 2010; Wang et al. 2011; Phillips et al. 2013; Slippers et al. 2013; Ko et al. 2023). The results consistently indicated the possible existence of multiple cryptic species in *L.theobromae*.

*Lasiodiplodiatheobromae* was first documented in cacao (Nowell 1923) as a morphological variable and multi-host ascomycete with predominantly asexual reproduction. Our previous study focusing on phylogenetic relations of species in *Botryosphaeriaceae* in Taiwan also supported the possibility of cryptic species and genetic variation within *Lasiodiplodia* (Ko et al. 2023). Notably, *L.theobromae* isolates were found to be paraphyletic, with some of the isolates grouping with *L.brasiliense* (Ko et al. 2023). This finding highlighted the ongoing challenges in accurately delineating species boundaries within this genus. Therefore, an in-depth genetic analysis is necessary to uncover hidden diversity further and clarify taxonomic uncertainties in *Lasiodiplodia*.

The generalist pathogen *L.theobromae* adapts to various hosts and infects different host tissues. However, true generalist pathogens are rare, as molecular studies often reveal cryptic species within what were once thought to be generalist species (Le Gac et al. 2007; Pintye et al. 2015; Beckstead et al. 2016). Although generalist plant pathogens can infect a wide range of hosts, they often comprise multiple strains that exhibit varying degrees of genetic differentiation, host preference or narrower host ranges. This has been observed in several studies on generalist plant pathogens (Fournier and Giraud 2008; Pintye et al. 2015; Walker et al. 2015; Feurtey et al. 2016; Menardo et al. 2017). This variability underscores their adaptive potential, where such pathogens can maintain broad host ranges while maintaining host-specific specialisations amongst certain strains, leading to differential impacts on plant fitness. The dynamic interplay between generalist tendencies and effective specialisation highlights their capability to adaptively infect various hosts, while also exerting context-dependent selective pressures (Benítez et al. 2013; Beckstead et al. 2016). In the case of *L.theobromae*, previous studies have predominantly reported low genetic differentiation amongst samples from various hosts or geographic regions (Burgess et al. 2003; Mohali et al. 2005; Shah et al. 2010; Begoude Boyogueno et al. 2012; Mehl et al. 2017). However, some research has detected weak genetic differentiation unrelated to geography and host (Burgess et al. 2003; Mohali et al. 2005; Begoude Boyogueno et al. 2012; Mehl et al. 2017; Rêgo et al. 2019).

Interestingly, despite *L.theobromae* being primarily considered an asexually reproducing organism, genetic diversity studies have yielded significantly varied results (Cardoso and Wilkinson 2008; Begoude Boyogueno et al. 2012; Sangeetha et al. 2012; Al-Sadi et al. 2013; Rêgo et al. 2019). These notable discrepancies in genetic diversity studies raise a crucial question: Are the effects of host species diversity and geographic distribution more influential than previously thought? To address this, examining the genetic structure of *L.theobromae* across various ecological contexts is crucial, especially considering the complexities of generalist pathogens. Given the inconclusive nature of current genetic differentiation patterns, a comprehensive study of *L.theobromae*’s genetic structure and its relationship with isolate origin, host and cultivars is necessary to understand this adaptable pathogen better.

In our previous study, four genetic markers were applied to elucidate phylogenetic relationships of *Lasiodiplodia* species and the closely-related species infected fruits in Taiwan (Ko et al. 2023). The four markers included intergenic spacer (ITS) of genomic rDNA, nuclear ribosomal small subunit (SSU), nuclear ribosomal large subunit (LSU), translation elongation factor 1-alpha (EF1), β-tubulin gene (TUB) and mitochondrial ribosomal small subunit (mtSSU). These markers provided appropriate resolution for the phylogenetic relationships of the investigated species. However, as mentioned, we observed potential host preferences in *Lasiodiplodia* species and possible cryptic species of *L.theobromae* that the four genetic markers may not adequately reveal. Genetic markers with higher intraspecific variation are necessary to uncover the species’ evolutionary mode and genetic differentiation.

In this study, we aim to use transferable microsatellites, which can be amplified using identical primer pairs across closely-related species, to address the questions mentioned above. Although microsatellite loci have been developed, based on the genome of *L.theobromae*, their “transferability” between *Lasiodiplodia* species and other members of the *Botryosphaeriaceae* remains limited (Burgess et al. 2003; Slippers et al. 2004; Cardoso and Wilkinson 2008; Baird et al. 2010; Nagel et al. 2020; Nagel et al. 2020). Therefore, this study aims to achieve three key objectives: (1) Evaluate the cross-species applicability of microsatellite markers within the *Botryosphaeriaceae*, including six species of genus *Lasiodiplodia* and two species of genus *Neofusicoccum* collected from important fruit crops in Taiwan; (2) Conduct a comprehensive genetic diversity and differentiation analysis while examining the degree of host or origin specificity to identify distinct genetic clusters; (3) Examine the demographic history of *Lasiodiplodia* species and assess the influence of gene flow on their evolutionary trajectory.

## ﻿Materials and methods

### ﻿*Lasiodiplodia* and other *Botryosphaeriaceae* Isolates from fruit crops in Taiwan

In this study, genomic DNA samples were employed from a comprehensive survey of *Lasiodiplodia* species infecting fruit crops in Taiwan (Ko et al. 2023). We obtained 209 genomic DNA samples from single spores of *Lasiodiplodia* and *Neofusicoccum* from various host plant families: *Myrtaceae* — *Syzygiumsamarangense* (wax apple), *Psidiumguajava* (guava) and *Syzygiumtaiwanicum*; *Anacardiaceae* — *Mangiferaindica* (mango); *Annonaceae* — *Annonasquamosa* (sugar apple); *Caricaceae* — *Caricapapaya* (papaya); *Malvaceae* — *Theobromacacao* (cocoa); *Boraginaceae* — *Cordiadichotoma*; *Zingiberaceae* — *Alpinia*; and *Musaceae* — *Musa* spp. (banana). Species identification was conducted using a multi-locus phylogenetic approach, employing the internal transcribed spacer (ITS) region, translation elongation factor 1-α (TEF1-α) and β-tubulin (TUB2) gene sequences. There were 145 *Lasiodiplodia* genomic DNA samples, including six species: 63 *L.theobromae*, 11 *L.brasiliensis*, 18 *L.hormozganensis*, 13 *L.pseudotheobromae*, 26 *L.rubropurpurea* and 14 *L.iranensis*. Additionally, 64 genomic DNA samples from other genera within the *Botryosphaeriaceae* family, including 12 *Neofusicoccummangiferae* and 52 *Neofusicoccumparvum* (Suppl. material 2: table S1).

### ﻿Microsatellite genotyping

Microsatellite markers amplified in 20 μl PCR mix containing 0.5 μl of template DNA, 2 μl of 10× reaction buffer, 2 μl of dNTP Mix (2 mM), 2 μl of each forward and reverse primer (2 mM), 0.5 μl *Taq* polymerase (0.2 U μl^-1^; Promega) and 11 μl sterile water. The gradient PCR reactions of initial denaturation of 94 °C for 5 min, followed by 35 cycles of the 45 s at 94 °C, 45 s at 50﻿–65 °C, the 50 s at 72 °C and set the final extension of 7 min at 72 °C were performed using the Labnet MultiGene 96-well Gradient Thermal Cycler (Labnet, Edison, New Jersey, USA).

After confirming the optimum annealing temperature (*T_a_*) for each primer, all samples performed the PCR amplification reaction. The PCR products were separated and assessed using 10% polyacrylamide gel electrophoresis (a mix of 30% acrylamide, 5× TBE buffer, 10% ammonium peroxydisulphate and tetramethyl ethylenediamine) performed in 1× TBE as the electrophoresis buffer at 70 V for 16 h, stained with ethidium bromide and visualised (used ethidium bromide) under UV light exposure. The alleles’ patterns and sizes were recorded digitally using Quantity One ver. 4.62 (Bio-Rad Laboratories, Hercules, California, USA).

### ﻿Assessment of transferability, gene diversity and recombination

Six *Lasiodiplodia* species and two *Neofusicoccum* species were isolated from various fruit trees in Taiwan. These species were used to evaluate the cross-species and cross-genus transferability of twenty-two microsatellite markers. PCR amplification of microsatellite markers was performed on twenty isolates from eight species, followed by fragment length analysis to assess transferability and polymorphisms under the previously described conditions. The polymorphism information content (PIC) for each primer was calculated using Cervus 3.0.7 to estimate allele variation (Kalinowski et al. 2007).

The allelic richness (*A_r_*) and private allelic richness (*A_p_*: the unique alleles number in a population) of *Lasiodiplodia* and *Neofusicoccum* species were calculated in HP-Rare (Kalinowski 2005), which is the fundamental parameter of genetic diversity. Poppr v.2.9.2, an R package, is convenient for analysing genetic data of mixed reproduction modes with sexual and clonal production (Kamvar et al. 2014). Consequently, this study employed Poppr v.2.9.2 to calculate Nei’s unbiased gene diversity (*H*exp) (Kamvar et al. 2014).

Assessing genotypic diversity is crucial for analysing the genetic structure of pathogen and microbial populations (Grünwald et al. 2003). We evaluated the multilocus genotype (MLG) diversity of *Lasiodiplodia* species by calculating various parameters, including the observed Multilocus genotypes (MLG). To account for differences in sample sizes, we estimated the number of expected MLG (eMLG) using the rarefaction method, which standardizes the comparison to the smallest sample size (Grünwald et al. 2003). Genotypic diversity parameters were also measured using the Shannon-Wiener Index of MLG diversity (*H*) (Shannon and Weaver 1949), Stoddart and Taylor’s Index of MLG diversity (*G*) (Stoddart and Taylor 1988), Simpson’s Index (Lambda) and Evenness (*E*_.5_) (Grünwald et al. 2003). The clonal fraction of *Lasiodiplodia* species was calculated as 1 − (number of MLGs / number of isolates), which is defined as the proportion of the fungal isolates sampled originating from asexual reproduction (Zhan et al. 2003).

Random association of alleles at different loci, leading to gametic equilibrium in populations, is one of the long-term consequences of genetic recombination. Therefore, analysis of fungal populations for potential clonal or mixed clonal and sexual reproduction relies on testing the null hypothesis of random mating by detecting linkage disequilibrium amongst loci (Milgroom 1996; McDonald and Linde 2002). We used Poppr v.2.9.2 to calculate the index of association (*I*_A_) and its standardised form (rbarD) to detect signatures of multilocus linkage. Significant deviation from the null model, which assumes no linkage amongst markers, was assessed using four permutation algorithms per population dataset (Brown et al. 1980; Smith et al. 1993; Burt et al. 1996; Milgroom 1996; Agapow and Burt 2001).

### ﻿Genetic differentiation and genetic groups analysis

We conducted a hierarchical analysis of molecular variance (AMOVA) using Arlequin v.3.5 (Excoffier and Lischer 2010) to evaluate the partitioning of genetic variance across different levels and assess the significance of *F*-statistics through 999 permutations. Additionally, we calculated genetic distances amongst *Lasiodiplodia* species, based on the proportion of shared alleles (*D*_PS_) using the microsatellite analyser (MSA) v.4.05 (Dieringer and Schlötterer 2003).

To comprehensively examine the genetic structure patterns and identify genetic groups, we employed multiple software tools for cross-validation. These included Principal Coordinate Analysis (PCoA), Bayesian-based analysis (Structure), Discriminant Analysis of Principal Components (DAPC) and GENELAND. These analyses were utilised to investigate the genetic structure of *Lasiodiplodia* species collected from various fruit plants across different regions in Taiwan.

To evaluate the relationships amongst isolates, we conducted a multivariate analysis using PCoA in GenAlEx v.6.5 (Peakall and Smouse 2012). This distance-based model utilises a pairwise genetic distance matrix derived from Jaccard’s similarity coefficient to visualise genetic affinities between samples.

Structure clustering analyses employ Bayesian clustering to classify individuals into source populations based on allele frequencies (Pritchard et al. 2000). This method utilises the Markov Chain Monte Carlo (MCMC) estimation to analyse the distribution of genetic variation and group individuals with similar characteristics. The algorithm assigns individuals to K ancestry clusters, estimates variant frequencies and re-assigns individuals under the assumptions of Hardy-Weinberg and linkage equilibrium (Hubisz et al. 2009; Porras-Hurtado et al. 2013). In our study, we implemented an admixture model with population identifiers. We calculated posterior probabilities of K clusters for each individual using 10^5^ burn-in periods and 10^6^MCMC replicates. To ensure robust results, we conducted twenty independent Markov chain runs. We performed the analysis on partitioned datasets to examine lower-level genetic structure. First, *Lasiodiplodia* species would be analysed separately to censor the degree of genetic composition admixed amongst *Lasiodiplodia* species. Second, the datasets of *L.theobromae* were analysed independently to check the cryptic genetic groups. We utilised Structure Harvester to evaluate likelihood values across multiple K values and iterations (Earl 2012). CLUMPP was employed to align multiple replicates of the chosen K cluster, addressing multimodality issues (Jakobsson and Rosenberg 2007). For a visual representation of the Structure output, we generated bar plots using the POPHELPER R package (Francis 2017).

While Structure is effective for analysing recent mixtures amongst differentiated groups, additional methodologies are required for comprehensive demographic and historical analysis (Falush et al. 2016; Novembre 2016). Considering the diverse reproductive modes and mating systems of fungi, which often deviate from random mating conditions, DAPC emerges as a more appropriate tool for analysing various organisms. This model-free multivariate approach for DAPC (Jombart et al. 2010) was implemented using R v.4.1.0 with the adegenet 2.1.3 package (Jombart 2008). DAPC integrates discriminant analysis (DA) with principal component analysis (PCA), ensuring uncorrelated variables and a reduced number of variables compared to analysed individuals. It employs a sequential K-means algorithm to infer complex genetic clusters without prior group information, making it suitable for potentially clonal pathogen populations. This method optimises the discrimination of individuals into pre-defined groups by maximising between-group variance, while minimising within-group variance (Jombart et al. 2010). In our analysis, we initially employed the find.clusters() method to identify clusters (with a maximum of 40 evaluation groups) and assign samples. The optimal group number (K) was determined, based on Bayesian Information Criterion (BIC) differences. We then verified the number of principal components retained in the DAPC analysis using the xvalDapc function with default parameters (Miller et al. 2020).

While the DAPC approach offers valuable insights, its linear combination of genotypes may not fully capture complex genetic structures with multimodal distributions and non-linear patterns (Sugiyama 2007; Qin et al. 2021). To complement this analysis, we employed GENELAND v.4.9.2 in R v.4.4.1 to delineate genetic boundaries and cross-validate results with other grouping methods (Guillot et al. 2005; Guillot 2008; Guillot et al. 2012). The Bayesian clustering algorithm implemented in the GENELAND is based on a spatial cluster model, assuming that populations are spatially separated with few gene flows. GENELAND implements a spatial cluster model that assumes populations are spatially distinct with few gene flows, incorporating geographical coordinates into its analysis. We conducted the analysis using a correlated allele frequencies model, executing ten independent MCMC runs (100,000 iterations, thinning = 100, burn-in = 10,000). The maximum number of populations (*K*) was set to 40, with the Poisson process rate and Poisson-Voronoi tessellation nuclei maxima set at 100 and 300, respectively. The optimal *K* value was identified, based on the run exhibiting the highest likelihood (posterior density).

### ﻿Estimation of multilocus SSR genotype evolutionary relationships

We constructed a Minimum Spanning Network of *Lasiodiplodia* species using multilocus SSR genotypes to visualise genetic relationships and investigate potential recombination events. We employed Poppr v.2.9.2 to define MLGs and calculated Bruvo’s distance, which is recommended for microsatellite data due to its stepwise mutation model. Bruvo’s distance incorporates microsatellite repeat numbers, with a distance value of 0.1 representing a single mutation step. This approach provides a robust framework for analysing genetic relatedness within the *Lasiodiplodia* species complex (Bruvo et al. 2004).

### ﻿Isolation-with-migration analyses

We used IMa3 (Hey et al. 2018) to analyse demographic parameters across six *Lasiodiplodia* species and distinct genetic groups of *L.theobromae*. Our methodology incorporated the hidden genealogy approach (-j0) for phylogenetic topology estimation and employed the Stepwise Mutation Model for microsatellite data analysis. All the present and their ancestral populations (species) had the effective population size parameter (denoted by *q*). Migration rate parameters (denoted by *m*) between all pairs of source and target populations were provided. The splitting times of (denoted by *t*) of internal nodes corresponding to each ancestral population were also estimated. We calibrated demographic quantities using a mutation rate range of 2.80 × 10^-6^–2.50 × 10^-5^ per year. The microsatellite mutation rate was estimated, based on the noncoding DNA mutation rate in filamentous *Ascomycetes* (1.12 × 10^-9^ and 1.00 × 10^-8^ substitutions/site/year) (Kasuga et al. 2002) and then multiplied by 2500 to yield average microsatellite mutation rate of 2.80 × 10^-6^–2.50 × 10^-5^ (Dettman and Taylor 2004). The analysis comprised two independent runs, each sampling 200,000 genealogies with a 10% burn-in period. To visualise the isolation-with-migration model results, we employed the IMfig programme (Hey 2010; Hey et al. 2018).

## ﻿Results

### ﻿Transferability and polymorphisms of microsatellite markers amongst species of *Botryosphaeriaceae*

Fungal samples were extensively collected from important fruit crops in Taiwan. Eight species in the *Botryosphaeriaceae* were identified, including six species of *Lasiodiplodia* and two species of *Neofusicoccum* (Suppl. material 2: table S1). We first examined the transferability of microsatellite markers developed from *L.theobromae* in previous studies amongst all eight species. Sixteen of 22 makers could be successfully amplified and showed polymorphism in *Lasiodiplodia* and *Neofusicoccum* species (Table 1). These 16 markers demonstrated moderate to high values of Polymorphism Information Content (PIC), ranging from 0.33 (*LAS0304*) to 0.92 (*LAS2122*) (Table 1). Several microsatellite markers showed unique sizes for specific *Lasiodiplodia* species (Suppl. material 2: table S2). For instance, 176 bp of *LAS2930* and 408 bp of *LAS2122* were unique to *L.pseudotheobromae* and *L.iranensis*. *L.rubropurpurea* exhibited unique allele sizes in two markers: *LAS2526* (386 bp, 392 bp) and *LAS1718* (256 bp) (Suppl. material 2: table S2). Notably, *LAS2324* failed to amplify in *L.pseudotheobromae*, providing another distinctive feature for species identification. Both unique allele sizes and the absence of amplification can aid in differentiating *Lasiodiplodia* species.

**Table 1.** Repeat motifs, primer sequences, fragment sizes (bp) based on *L.theobromae*, optimised annealing temperatures (*Ta*), polymorphism information content (PIC) and *F_ST_* values of 16 polymorphic microsatellite loci used in this study. *p < 0.001.

Primer	Primer sequence (5’ to 3’)	Repeat motif	Fragment size (bp)	*Ta* (°C)	PIC	*F_ST_*
*LAS1314*	F: 5’-GAGTTGTTAGTGCGGGCGCC-3’	A_5_(GA)_3_(GAAGAAA)_2_(GA)_3_ A_5_(CGG)_3_	317	63	0.43	0.32*
	R: 5’-GCAGCCCCACAATTCACCAG-3’					
*LAS1516*	F: 5’-GCCAGATCCGTGCCCACTG-3’	(CT)_3_(AG)_3_-TCTCTT_7_	335	63	0.89	0.23*
	R: 5’-CATGCAGAGGTCGCAAAGTG-3’					
*LAS2122*	F: 5’-GGAAGATGATGGGATGGTTGC-3’	(CA)_5_T_6_(GCT)_3_G_7_T_8_	387	58	0.92	0.26*
	R: 5’-GTACAAGAACGAACTCCGGGT-3’					
*LAS2728*	F: 5’-CGAACAGGGTTTCGTGACGT-3’	(GA)_3_(GAC)_4_(TTC)_3_(CG)_4_(TCGC)_3_(GT)_7_(GA)_3_(CTCTCG)_3_	462	58	0.81	0.31*
	R: 5’-CTCATATCTCGCCGGTTGCC-3’					
*LAS3536*	F: 5’-GGCATCACAACGACCAACCC-3’	(GCTT)_10_(GGA)_5_(CGT)_4_(GCT)_5_	379	63	0.87	0.31*
	R: 5’-GCGAGAGTCGCAAGTACAGC-3’					
*LAS0304*	F: 5’-GACTCATTCACGGTCTCATGG-3’	T_5_(CT)_2_CA(CT)_5_G_5_AG_4_(GT)_4_	361	57	0.33	0.24*
	R: 5’-GTGGAGCGGAACTGTCTGCT-3’					
*LAS1718*	F: 5’-GATCTTCCAGCTCTTCGGCC-3’	Sequence rich in A repeats	254	57	0.88	0.34*
	R: 5’-GACACTGCAGTAGGTTAGCGG-3’					
*LAS3334*	F: 5’-GCTCCGTTGCGCAAGAGCAG-3’	(CCCTTTCCTCTTCTTT)(GCT)_5_	276	57	0.87	0.29*
	R: 5’-GTCTTGTCTGAACGCCTTCGC-3’					
*LAS3738*	F: 5’-GGTTACTCGACGATGATCTCC-3’	(GATGTGTGT)_4_(GTGTTGGTGTGTTGTGT)	135	57	0.89	0.20*
	R: 5’-CAGTCACTTACCACGACACC-3’					
*LAS2930*	F: 5’-GACGAGGTCAAGGGCGACA-3’	(CGA)_3_(CAA)_7_(GCA)_3_	191	52	0.84	0.47*
	R: 5’-CCTCCATGTCGGATTCCTTG-3’					
*LAS2526*	F: 5’-GTATTGCAAGGTGAGCAAGAG-3’	(GC)_7_(CA)_11_(CA)_4_T_7_	433	55	0.64	0.80*
	R: 5’-GTAGATGGCGTGTATCATCCT-3’					
*LAS3132*	F: 5’-GGGTGTGTTACCCGAATCAG-3’	(GT)_4_	437	56	0.89	0.18*
	R: 5’-CGCCATTTGCTTGCCTACAGC-3’					
*LAS2324*	F: 5’-CAAAGCGATTGTACGCGGGT-3’	(CT)_3_(AGTG)_8_(GGGCT)_7_T_13_	456	56	0.88	0.34*
	R: 5’-CACGGTTGGACCAACCCGTG-3’					
*LAS 01*	F: 5’-GAGGGTTTTGTGCTCCATGT-3’	(CA)_6_	202	57	0.61	0.51*
	R: 5’-GGAAAACGGTGGTCAAAGAA-3’					
*LAS 08*	F: 5’-CTCGTTAGGAAGGAAAGCAT-3’	(GGT)_7_	188	58	0.74	0.39*
	R: 5’-GAACTATCCCCGCATCTACT-3’					
*LAS 09*	F: 5’-GGGAAAATAAAATGGTCTGG-3’	(GA)_9_	143	58	0.67	0.36*
	R: 5’-GAAACCCTTGTTCCATGC-3’					

### ﻿Assessment of genetic diversity and inbreeding in eight species

A total of 216 alleles were identified from 16 microsatellite loci and 144 multilocus genotypes (MLGs) were identified amongst 146 *Lasiodiplodia* samples. The allelic richness (*A_r_*) and private allelic richness (*A_p_*) were highest in *N.parvum* (*A_r_*: 6.63; *A_p_*: 2.52) and lowest in *L.brasiliensis* (*A_r_*: 2.98; *A_p_*: 0.05) (Suppl. material 2: table S3). Amongst the six *Lasiodiplodia* species, *L.rubropurpurea* exhibited the highest allelic richness and private allelic richness (*A_r_*: 5.63; *A_p_*: 1.45).

While MLG numbers varied across species, expected MLG counts (eMLG) remained relatively consistent. The highest MLG diversity was observed in *L.theobromae* (*H*: 4.14; *G*: 63), while the lowest was observed in *L.brasiliensis* (*H*: 2.27; *G*: 9.31). Nei’s unbiased gene diversity (*H*exp) spanned from 0.71 (*L.rubropurpurea*) to 0.42 (*L.brasiliensis*). Evenness (*E*_.5_) values were consistently high across species (0.96–1.00) (Table 2).

**Table 2.** Parameters of genotype diversity in six *Lasiodiplodia* species, based on microsatellite data generated by the poppr() function.

Species	N	MLG	eMLG	SE	*H*	*G*	lambda	*E.*_5_	*H*exp	Clonal fraction	*I*_A_	rbarD
***L.brasiliensis***	11	10	10.00	0.00	2.27	9.31	0.89	0.96	0.42	0.09	4.52*	0.31*
***L.hormozganensis***	18	17	10.60	0.48	2.81	16.20	0.94	0.97	0.70	0.06	1.79*	0.12*
***L.pseudotheobromae***	13	13	11.00	0.00	2.56	13.00	0.92	1.00	0.49	0.00	0.78*	0.06*
***L.rubropurpurea***	27	27	11.00	0.00	3.3	27.00	0.96	1.00	0.71	0.00	0.43*	0.03*
***L.theobromae***	63	63	11.00	0.00	4.14	63.00	0.98	1.00	0.69	0.00	0.84*	0.06*
***L.iranensis***	14	14	11.00	0.00	2.64	14.00	0.93	1.00	0.64	0.00	0.97*	0.07*
**Total**	146	144	11.00	0.10	4.96	142.11	0.99	0.99	0.78	0.01	0.49*	0.03*

N: Number of isolates; MLG: Multilocus genotypes (MLG) observed; eMLG: The number of expected MLG at the smallest sample size (≥ 10) based on rarefaction; SE: Standard error based on eMLG; *H*: Shannon-Wiener Index of MLG diversity; *G*: Stoddart and Taylor’s Index of MLG diversity; Lambda: Simpson’s Index; *E.*_5_: Evenness; *H*exp: Nei’s unbiased gene diversity; *I*_A_: The index of association; RbarD: The standardised index of association; *Significant at *p* < 0.01.

All six *Lasiodiplodia* species exhibited low clonal fractions and high genetic diversity. To detect evidence of recombination, the index of association (*I*_A_) and its standardised index of association (rbarD) were calculated, showing low, yet statistically significant values across all *Lasiodiplodia* species. *L.brasiliensis* demonstrated the highest values (*I*_A_: 4.52; rbarD: 0.31), whereas *L.rubropurpurea* exhibited the lowest (*I*_A_: 0.43; rbarD: 0.03). The null hypothesis of the random mating was rejected by detecting the linkage disequilibrium of alleles (Table 2).

### ﻿Species delineation and genetic divergence were observed in *Lasiodiplodia* species amongst host fruit plants

Since different evolutionary scenarios have been proposed for *Lasiodiplodia* in literature, we performed several analyses to elucidate its evolutionary pattern.

PCoA demonstrated that the microsatellite markers grouped *Lasiodiplodia* species into three major clusters (Suppl. material 2: fig. S1A). The first cluster included *L.pseudotheobromae* and *L.iranensis*. The second cluster consisted mostly of *L.rubropurpurea*, with three samples from *L.theobromae*. The third cluster contained *L.hormozganensis*, most samples of *L.theobromae* and *L.brasiliensis*.

Next, we investigated whether genetic variations in fungal isolates correlated with the host species or infection sites. The results revealed that genetic groupings had a clear association with host plants for each *Lasiodiplodia* species [Suppl. material 2: fig. S1B]. For instance, wax apple isolates formed distinct clusters in *L.hormozganensis*, *L.brasiliense*, *L.rubropurpurea* and *L.theobromae*. Similarly, mango isolates clustered distinctly in *L.hormozganensis*, *L.pseudotheobromae*, *L.brasiliense* and *L.theobromae*. Additionally, *L.hormozganensis* isolates from bananas and *L.theobromae* isolates from cocoa and papaya formed their own distinct clusters (Suppl. material 2: fig. S1B). However, no significant genetic grouping was observed between different infection sites (fruits and stems) (Suppl. material 2: fig. S1B). For example, while *L.brasiliensis* and *L.rubropurpurea* in mango showed distinct clustering based on host plants, they showed no differentiation based on infection site. Similarly, *L.theobromae* isolates from fruits and stems showed substantial genetic overlap in their groupings (Suppl. material 2: fig. S1B).

Significant genetic differentiation amongst species was identified in *Lasiodiplodia* species, with *F_ST_* values ranging from 0.07 to 0.48 (Suppl. material 2: table S4). The lowest genetic differentiation was observed between *L.hormozganensis* and *L.theobromae* (*F_ST_* = 0.07), while the highest was between *L.brasiliensis* and *L.pseudotheobromae* (*F_ST_* = 0.48) (Suppl. material 2: table S4). Furthermore, analysis of genetic distances, based on shared alleles, revealed values ranging from 0.40 to 0.77, with the lowest *D*_PS_ value amongst *L.hormozganensis*, *L.theobromae* and *L.brasiliensis*, while the highest was between *L.brasiliensis* and *L.rubropurpurea* (Suppl. material 2: table S4).

Analysis of genetic differentiation amongst *Lasiodiplodia* species across various host groups revealed significant differentiation within *Lasiodiplodia* species, with all *F_ST_* values exceeding 0.1. For *L.theobromae*, most host groups showed significant genetic differentiation, except for sugar apple, guava and mango groups, which displayed non-significant differentiation (Table 3). For example, the genetic differentiation of *L.brasiliensis* between wax apple and mango is significant (*F_ST_* = 0.7), even greater than the differences between *Lasiodiplodia* species (Table 3 and Suppl. material 2: table S4).

**Table 3.** List of pairwise genetic distance values (*F_ST_*) values amongst different host species of *Lasiodiplodia* species. Bold letters indicated that the data were significant (Significance Level = 0.05).

	LBR		LHO					LPSE			LRU				LTH								LIR			
	SS	MI	SS	PG	MI	AS	MB	SS	PG	MI	SS	PG	MI	C	SS	PG	MI	CP	AS	TC	CD	AL	PG	MI	AS	TC
LBR(SS)																										
LBR(MI)	**0.7**																									
LHO(SS)	**0.6**	0.8																								
LHO(PG)	**0.6**	**0.7**	0.8																							
LHO(MI)	**0.6**	0.5	**0.7**	**0.5**																						
LHO(AS)	**0.5**	0.4	**0.5**	**0.5**	**0.4**																					
LHO(MB)	**0.7**	0.7	**0.8**	0.8	**0.6**	**0.4**																				
LPSE(SS)	**0.6**	0.5	0.7	0.6	**0.5**	**0.4**	0.5																			
LPSE(PG)	**0.7**	**0.6**	**0.7**	**0.6**	**0.5**	**0.5**	**0.6**	**0.3**																		
LPSE(MI)	**0.7**	0.7	**0.8**	**0.7**	**0.6**	**0.5**	0.7	0.3	**0.4**																	
LRU(SS)	**0.5**	**0.4**	**0.5**	**0.5**	**0.4**	**0.3**	**0.5**	**0.4**	**0.5**	**0.5**																
LRU(PG)	**0.5**	**0.4**	**0.5**	**0.5**	**0.4**	**0.3**	**0.4**	**0.4**	**0.4**	**0.4**	**0.2**															
LRU(MI)	**0.7**	0.7	0.8	0.7	0.6	**0.5**	0.6	0.4	**0.5**	0.6	**0.3**	0.2														
LRU(C)	0.7	0.7	0.9	0.8	0.6	0.5	0.8	0.5	0.6	0.7	0.3	0.3	0.5													
LTH(SS)	**0.3**	**0.4**	**0.4**	**0.4**	**0.4**	**0.3**	**0.4**	**0.3**	**0.4**	**0.4**	**0.3**	**0.4**	**0.4**	**0.4**												
LTH(PG)	**0.4**	**0.3**	**0.4**	**0.3**	**0.3**	**0.2**	**0.4**	**0.3**	**0.4**	**0.4**	**0.3**	**0.3**	**0.3**	0.4	**0.2**											
LTH(MI)	**0.4**	0.3	**0.5**	**0.4**	**0.3**	**0.3**	**0.4**	**0.4**	**0.5**	**0.5**	**0.4**	**0.4**	**0.5**	0.5	**0.3**	**0.2**										
LTH(CP)	**0.5**	**0.5**	**0.5**	**0.6**	**0.5**	**0.2**	**0.5**	**0.5**	**0.5**	**0.6**	**0.4**	**0.4**	**0.5**	0.5	**0.4**	**0.3**	**0.4**									
LTH(AS)	**0.5**	0.3	**0.5**	**0.5**	**0.4**	0.2	0.4	**0.4**	**0.5**	**0.5**	**0.4**	**0.3**	0.5	0.5	**0.2**	0.1	0.2	**0.3**								
LTH(TC)	**0.5**	**0.5**	**0.5**	**0.6**	**0.5**	**0.3**	**0.5**	**0.5**	**0.6**	**0.6**	**0.4**	**0.4**	0.6	0.6	**0.3**	**0.3**	**0.3**	**0.3**	**0.3**							
LTH(CD)	0.6	0.8	0.9	0.8	0.5	0.4	0.8	0.6	0.6	0.7	0.5	0.4	0.7	0.9	0.3	0.3	0.4	0.4	0.3	0.4						
LTH(AL)	0.6	0.7	0.8	0.8	0.5	0.2	0.8	0.5	0.6	0.7	0.4	0.4	0.7	0.8	0.3	0.3	0.3	0.4	0.3	0.3	0.9					
LIR(PG)	0.7	0.7	0.9	0.7	0.5	0.4	0.8	0.4	0.5	0.6	0.4	0.4	0.7	0.8	0.3	0.3	0.4	0.5	0.4	0.6	0.9	0.9				
LIR(MI)	**0.5**	**0.4**	**0.5**	**0.4**	**0.4**	**0.3**	**0.4**	**0.2**	**0.3**	**0.3**	**0.3**	**0.3**	**0.4**	0.4	**0.3**	**0.3**	**0.3**	**0.4**	**0.3**	**0.4**	0.3	0.3	0.2			
LIR(AS)	**0.7**	0.7	0.8	0.8	**0.6**	**0.5**	0.8	**0.5**	0.5	**0.6**	**0.5**	**0.5**	0.7	0.8	**0.4**	**0.4**	**0.5**	**0.5**	0.5	**0.6**	0.8	0.8	**0.7**	0.3		
LIR(TC)	**0.8**	0.8	0.9	0.8	**0.7**	**0.6**	**0.8**	0.5	**0.6**	**0.7**	**0.6**	**0.5**	0.8	0.9	**0.5**	**0.5**	**0.5**	**0.6**	**0.6**	**0.7**	0.9	0.9	**0.8**	0.3	0.8	

Note: [Species code] LTH: *L.theobromae*; LBR: *L.brasiliensis*; LHO: *L.hormozganensis*; LPSE: *L.pseudotheobromae*; LRU: *L.rubropurpurea*; LIR: *L.iranensis*. [Host species code] SS: *Syzygiumsamarangense*; PG: *Psidiumguajava*; MI: *Mangiferaindica*; CP: *Caricapapaya*; AS: *Annonasquamosa*; TC: *Theobromacacao*; MB: *Musabasjoo*; C: unknown; AL: *Alpinia*; CD: *Cordiadichotoma*.

Examination of accumulated genetic variation across different classes indicated that variation was primarily distributed within populations (43.75%), amongst populations within *Lasiodiplodia* species (29.84%) and amongst species (20.59%). Notably, the majority of genetic variation was observed within populations (57.86%) in *L.theobromae* (Suppl. material 2: table S5). The fixation index was significantly high amongst *Lasiodiplodia* species (*F_CT_* = 0.21), amongst populations within species (*F_SC_* = 0.38) and within populations (*F_ST_* = 0.57 and *F_ST_* = 0.37), indicating significant genetic differentiations at all levels (Suppl. material 2: table S5).

### ﻿Genetic structures of six *Lasiodiplodia* species

To further elucidate the genetic structure of six *Lasiodiplodia* species, we conducted DAPC, Structure and GENELAND analyses to determine optimal clustering and genetic structure patterns.

**Figure 1.**
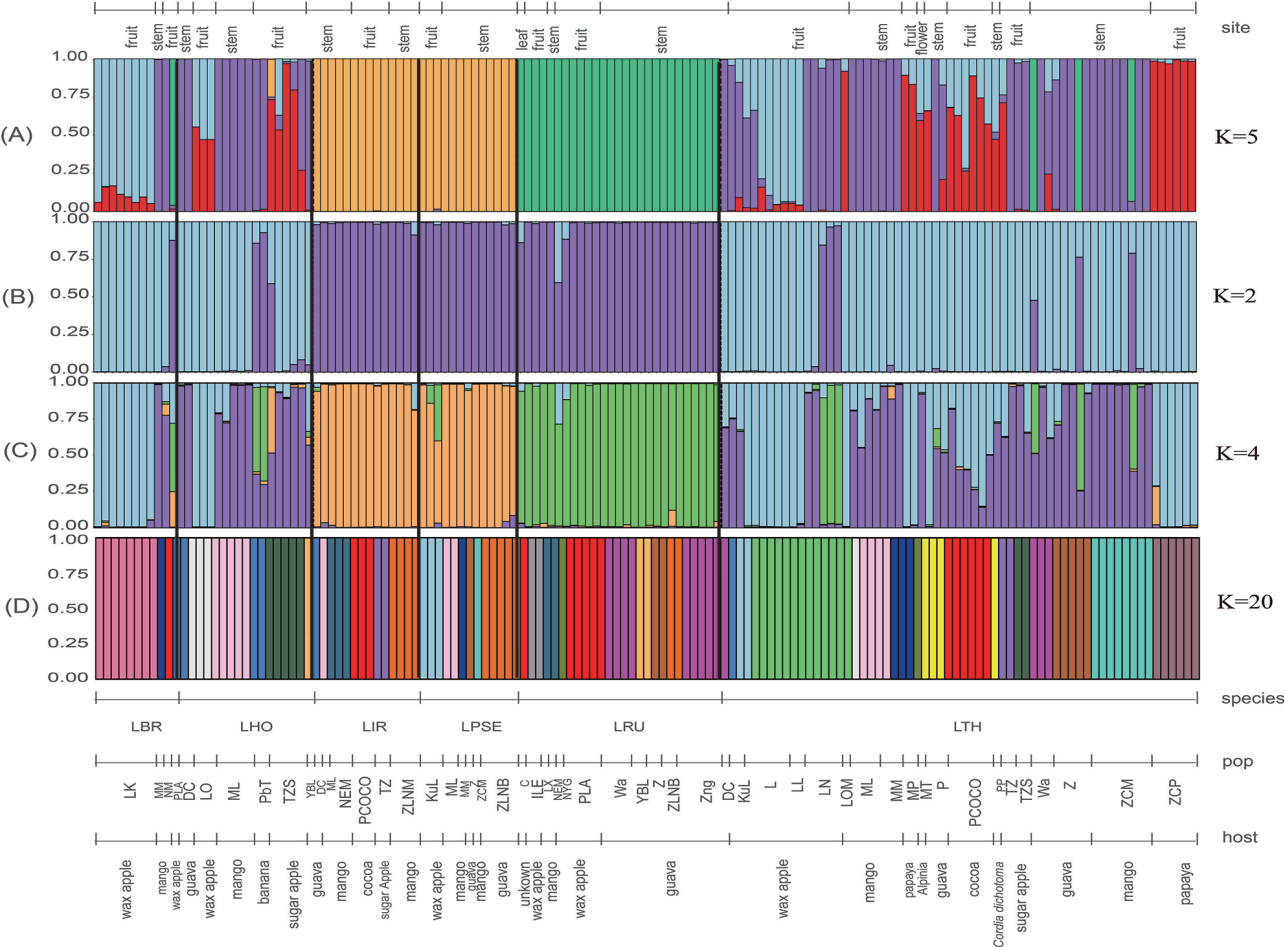
Bar plots for the genetic structure of *Lasiodiplodia* species, based on microsatellite data generated by (**A**) DAPC, (**B, C**) Structure and (**D**) GENELAND. A vertical colour line represents each individual and the same colour indicates that the individual belongs to the same cluster. Black lines separate different species. The species codes are as follows: LBR: *L.brasiliense*, LHO: *L.hormozganensis*, LPSE: *L.pseudotheobromae*, LRU: *L.rubropurpurea*, LTH: *L.theobromae*, LIR: *L.iraniensis*.

DAPC analysis identified an optimal clustering solution of five groups (K = 5), with 20 principal components retained as recommended by the xvalDAPC function. The two primary discriminant analysis axes explained 73.6% of the total variance, with PC1 accounting for 45.12% and PC2 for 28.48% (Suppl. material 2: figs S2A, S3A). The analysis with K = 5 revealed that *L.rubropurpurea* (LRU) formed a distinct cluster, while *L.pseudotheobromae* (LPSE) and *L.iranensis* (LIIR) grouped into a unique cluster as well (Fig. 1A and Suppl. material 2: fig. S3A). In contrast, *L.theobromae* (LTH), *L.hormozganensis* (LHO) and *L.brasiliensis* (LBR) exhibited diverse genetic compositions, warranting further analysis to uncover potential cryptic genetic groups (Fig. 1A).

Structure analysis identified the optimal cluster is two clusters (K = 2), followed by twelve (K = 12) and three (K = 3) clusters amongst six *Lasiodiplodia* species and two *Neofusicoccum* species (Suppl. material 2: figs S2C, S4). At K = 12, the analysis effectively distinguished between *Lasiodiplodia* and *Neofusicoccum* species (Suppl. material 2: fig. S4). The analysis demonstrated high efficiency in discriminating amongst *Botryosphaeriaceae* species using the microsatellite markers. The analysis also revealed partial intraspecies variations linked to host differences in *L.theobromae*, *L.rubropurpurea*, *L.brasiliensis* and *L.hormozganensis*, particularly from wax apple, guava and mango (Suppl. material 2: fig. S4).

Structure analysis of six *Lasiodiplodia* species independently revealed that the optimal number of clusters was two (K = 2), followed by four (K = 4) (Fig. 1B, C, Suppl. material 2: fig. S2D, table S6). At K = 4, the clustering pattern demonstrated substantial concordance with DAPC analysis (K = 5), where *L.rubropurpurea* formed a distinct cluster and *L.pseudotheobromae* and *L.iranensis* grouped into a separate cluster. However, DAPC analysis exhibited superior resolution in detecting fine-scale cryptic genetic groups within *L.hormozganensis*, *L.theobromae* and *L.brasiliensis* (Fig. 1A, C). GENELAND analysis identified K = 20 as the optimal number of clusters based on the highest posterior density (Fig. 1D and Suppl. material 2: fig. S2F). The genetic structure of the *Lasiodiplodia* species showed no correlation with geographic distribution. Instead, the genetic structure pattern revealed distinct host-associated differentiation amongst *Lasiodiplodia* species (Fig. 1D).

### ﻿Intraspecific cryptic genetic structure in *Lasiodiplodiatheobromae*: delineation and characterisation of cryptic genetic groups

Given the substantial evidence of intraspecific genetic structure within *L.theobromae*, we conducted comprehensive analyses of its genetic structure using multiple analytical approaches: DAPC, Structure and GENELAND. To investigate potential drivers of genetic differentiation, we examined the genetic structure patterns in relation to two biological factors: host species affiliation and infection site.

**Figure 2.**
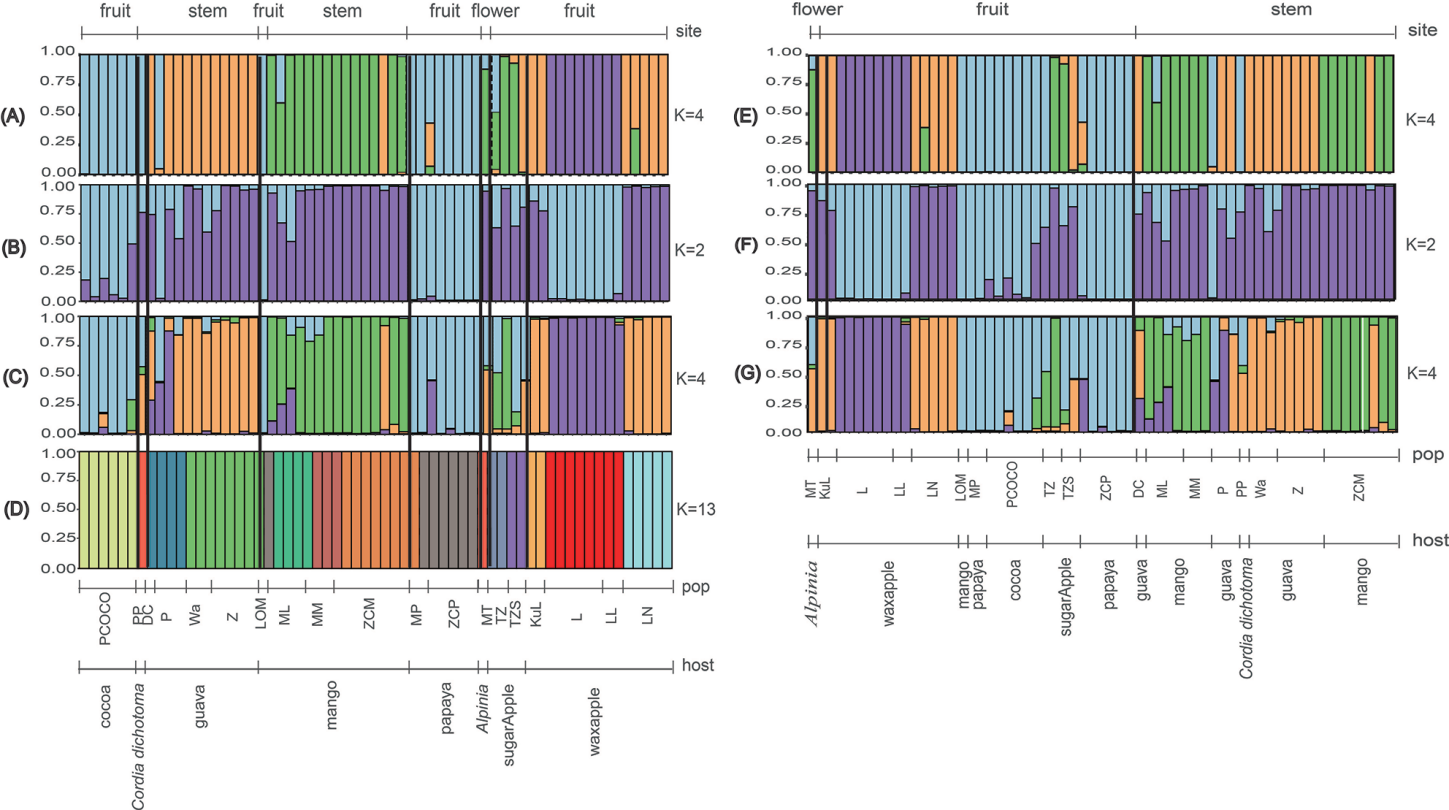
Genetic structure analysis of *Lasiodiplodiatheobromae*, based on microsatellite data. Bar plots representing the genetic structure of *L.theobromae* populations as inferred by different clustering methods: (**A** and **E**) DAPC analysis; (**B, C, F** and **G**) Structure analysis; and (**D**) GENELAND analysis. Each vertical line represents an individual, with colours indicating cluster membership. Panels A-D show clustering by host species, while panels E-G show clustering by infected sites. The Structure analysis is presented with two different optimal K values (**B, C** and **F, G**) to illustrate potential substructure.

For the DAPC analysis of *L.theobromae*, the optimal cluster number was determined to be K = 4, with 20 principal components retained as recommended by the xvalDAPC function. The two primary discriminant analysis axes explained 85.28% of the total variance, with PC1 accounting for 46.72% and PC2 for 38.56% (Suppl. material 2: fig. S2B, S3B). Based on the DAPC analysis with K = 4, we evaluated the genetic differentiation in relation to host species and infection sites. The results revealed that genetic differentiation was associated with host species differences (Fig. 2A), but showed no clear relationship with infection site variation (Fig. 2E). When examining the genetic composition of isolates arranged by host species, host-specific clustering was evident, with isolates from cocoa and papaya forming a single cluster, while those from mango and sugar apple predominantly grouped together. Wax apple isolates split into two genetic groups and guava isolates shared genetic composition with a subset of wax apple isolates, except for one sample (Fig. 2A).

**Figure 3.**
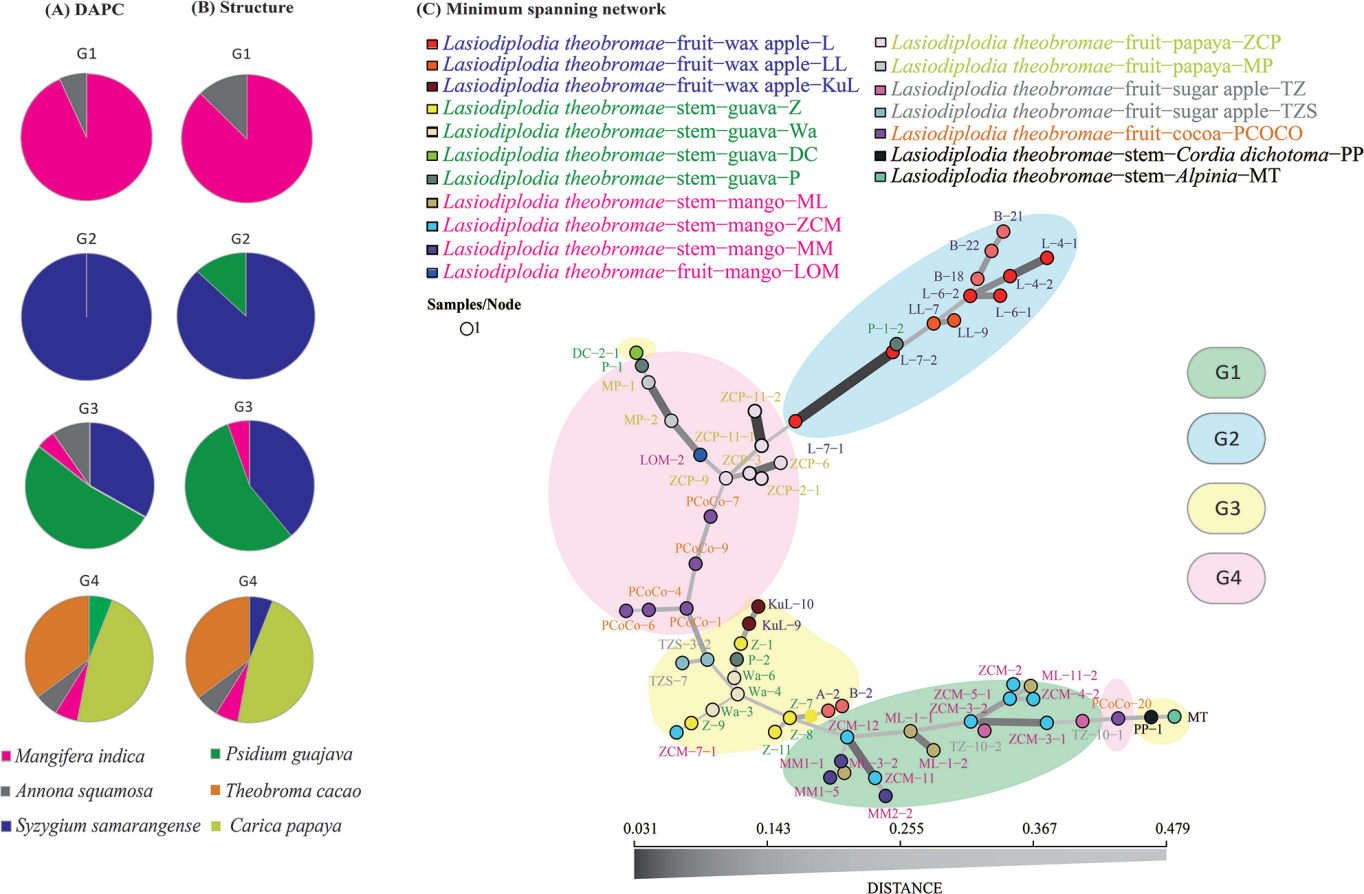
Proportional representation of four genetic groups in *Lasiodiplodiatheobromae* and their corresponding minimum spanning network. **A, B** the pie charts display the host species’ proportion across four genetic groups of *L.theobromae* inferred by DAPC and structure analysis **C** minimum spanning network illustrating the genetic relationships amongst the four genetic groups calculated via Bruvo’s distance, based on MLGs.

DAPC classification results revealed distinct genetic compositions within *L.theobromae*, with four genetic groups (G1-G4) showing minimal admixture (Fig. 3A and Suppl. material 2: fig. S3B). To examine host-specific associations, we evaluated the distribution of host species across these genetic groups. The analysis revealed clear host-specific patterns: G1 primarily consisted of mango isolates, with a few sugar apple samples; G2 exclusively contained wax apple isolates; G3 and G4 included isolates from multiple host species, with cocoa and papaya isolates appearing uniquely in G4 (Fig. 3A). To assess potential morphological distinctions, *L.theobromae* isolates were cultured on PDA at 25 °C for four weeks to evaluate phenotypic differences amongst genetic groups. Despite observable variations in culture morphology amongst the four genetic groups (Suppl. material 2: fig. S5), these morphological differences alone were insufficient to distinguish between genetic groups reliably.

Both Structure and GENELAND analyses provided complementary evidence for host-associated genetic differentiation in *L.theobromae*. Structure analysis identified four optimal clusters (K = 4) that aligned with DAPC findings (Suppl. material 2: fig. S2E, table S6 and Figs 2A, C, 3A, B), while GENELAND analysis revealed finer-scale subdivision with thirteen clusters (K = 13) (Fig. 2D and Suppl. material 2: fig. S2G). Minor variations in group composition were observed, particularly for guava and sugar apple samples (Figs 2A, C, 3A, B). Both analyses showed that genetic differentiation was significantly associated with host species (Fig. 2B–D), but independent of infection site differences (Fig. 2E–G). Notably, the genetic structure showed no correlation with geographical distribution, as evidenced by the extensive overlap of genetic groups across sampling locations, particularly in wax apple and guava populations (Fig. 2D). As DAPC has proven reliable for analysing fungi with complex mating systems, we conducted subsequent demographic estimations using the four genetic groups (G1-G4) identified through DAPC analysis.

### ﻿Multilocus genotype (MLG) evolutionary relationships

To investigate the phylogenetic relationships amongst different species and elucidate the evolutionary relationships between the four genetic groups within *L.theobromae*, we conducted comprehensive phylogenetic analyses. The minimum spanning network revealed that *L.theobromae* samples exhibited high polymorphism and complex genetic relationships amongst isolates. *Lasiodiplodiapseudotheobromae* and *L.iranensis* showed the closest phylogenetic relationships (Suppl. material 2: fig. S3C). This pattern was similar to the Neighbour-Joining relationship derived from the combined sequence dataset by Ko et al. (2023) (Ko et al. 2023).

The minimum spanning network analysis of *L.theobromae* revealed distinct and well-defined clustering patterns when colour-coded by the four genetic groups (G1-G4). The network showed clear genetic group segregation, with only four isolates clustering outside their designated groups (Fig. 3C). G1, primarily composed of mango isolates, exhibited a closer phylogenetic relationship to G3, consisting mainly of guava isolates. On the other hand, G4, comprising mostly papaya and cocoa isolates, showed a more distant phylogenetic relationship to G2, which included wax apple isolates, than to G1 and G3 (Fig. 3C).

Network analysis of the remaining five *Lasiodiplodia* species revealed significant host-associated genetic differentiation despite their comparatively limited sample sizes relative to *L.theobromae* (Suppl. material 2: fig. S6). This host-specific genetic structuring was particularly pronounced in *L.rubropurpurea* and *L.hormozganensis*, where distinct genetic clusters were observed among isolates from wax apple, mango and guava hosts Suppl. material 2: fig. S6B, E).

### ﻿Demographic parameter estimation

**Figure 4.**
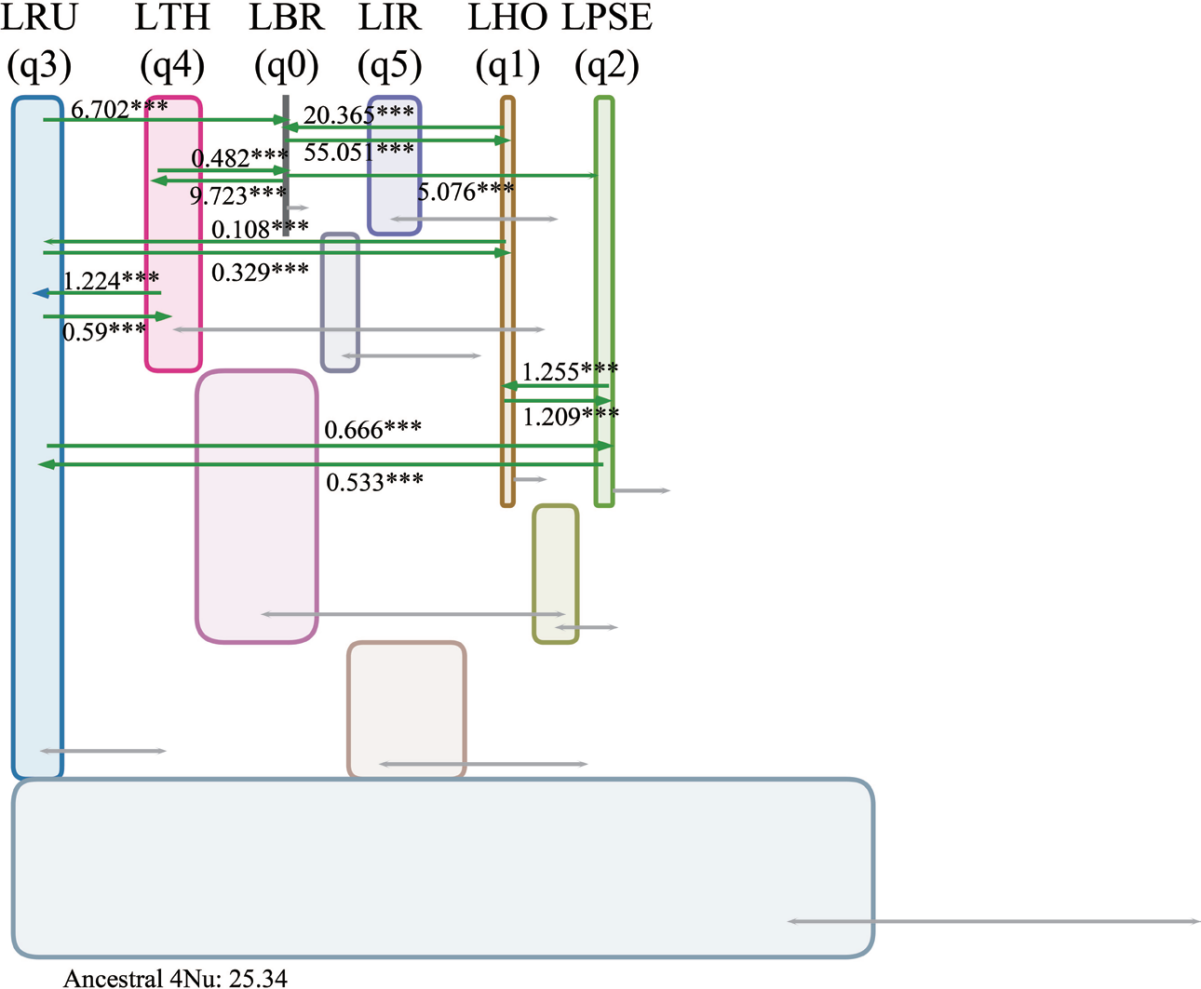
Representation of the Isolation with Migration model generated by IMa3 and the IMfig programme for the six *Lasiodiplodia* species. The corresponding phylogenetic topology is presented in Suppl. material 2: fig. S7 (**A** and **D**), with phylogeny depicted as a set of hierarchical boxes. Ancestor boxes are located between their corresponding descendants. The width of each box is proportional to the estimated effective population size (*Ne*), with grey arrows indicating the 95% confidence interval of the *Ne* value. Green arrows show migration direction (*p < 0.05, **p < 0.01, * ** *p* < 0.001). Values above the green arrows represent unscaled migration rate parameters. The species codes are as follows: 0: *L.brasiliense*, 1: *L.hormozganensis*, 2: *L.pseudotheobromae*, 3: *L.rubropurpurea*, 4: *L.theobromae*, 5: *L.iraniensis*.

Isolation-with-Migration models were employed to evaluate gene flow rates and effective population sizes. Amongst the six *Lasiodiplodia* species analysed, *L.theobromae* exhibited the highest effective population size, while *L.brasiliensis* demonstrated the lowest. Notably, *L.theobromae*’s ancestral effective population size approximated its current size. *L.iranensis* presented the second-highest effective population size, significantly larger than its ancestral size (Fig. 4, Suppl. material 2: table S7, fig. S8A, B).

Inter-species migration rates amongst *Lasiodiplodia* species were found to be exceptionally low. The highest migration rate was observed between *L.brasiliensis* and *L.hormozganensis* (Fig. 4, Suppl. material 2: table S7, fig. S8C). *L.theobromae* demonstrated minimal or no migration with other *Lasiodiplodia* species, except for minor gene flow with *L.rubropurpurea* and *L.brasiliensis*. It is worth noting that the migration rate from *L.brasiliensis* to *L.theobromae* exceeded that in the opposite direction (Fig. 4 and Suppl. material 2: table S7).

Examination of the four genetic groups (G1-G4) within *L.theobromae* revealed that G3 and G4 possessed higher effective population sizes compared to G1 and G2. Interestingly, the current effective population size of G2 was significantly larger than its ancestral size (Suppl. material 2: fig. S7D, E, table S8). Migration rates amongst these four genetic groups were determined to be low (Suppl. material 2: fig. S7F, E, table S8).

## ﻿Discussion

### ﻿Strategies for the rapid identification of *Lasiodiplodia* species with high-efficiency microsatellite markers

Rapid and accurate identification of *Lasiodiplodia* and *Neofusicoccum* species is crucial in plant pathology. These two genera frequently co-occur in the field, but traditional identification methods using mycelial culture morphology are unreliable because of similar colony characteristics and the time-consuming process of spore formation. This situation emphasises the need for molecular markers that enable swift and precise species identification. Although microsatellite markers are widely used in population genetics, they have limitations at higher taxonomic levels, especially regarding cross-genus transferability. Caution is therefore warranted when making broad phylogenetic inferences solely from microsatellite data.

Despite these constraints, transferable microsatellite markers remain invaluable for investigating genetic diversity and evolutionary patterns amongst closely related pathogens. Our research identified sixteen polymorphic microsatellite loci that efficiently distinguish multiple *Lasiodiplodia* species and differentiate them from *Neofusicoccum* at an early culture stage. While microsatellite-based markers may not fully resolve more distantly related taxa across the entire *Botryosphaeriaceae*, they remain invaluable for inferring genetic diversity patterns, revealing population structures and detecting cryptic lineages within closely related *Lasiodiplodia* species.

This finding enhances the markers’ utility in distinguishing between different *Lasiodiplodia* species and *Neofusicoccum*, an aspect not previously examined (Burgess et al. 2003; Begoude Boyogueno et al. 2012; Mehl et al. 2013; Rêgo et al. 2019). Our results demonstrate that careful marker selection enables efficient identification and preliminary discrimination amongst closely related *Lasiodiplodia* and *Neofusicoccum* species, providing a foundation for more comprehensive phylogenetic and epidemiological studies.

### ﻿Genetic diversity and recombination of *Lasiodiplodia* species from fruit crops in Taiwan

A comprehensive analysis of the fine-scale genetic diversity and structure of *L.theobromae* is essential for understanding its adaptive and evolutionary potential. Our study of six *Lasiodiplodia* species found that *L.rubropurpurea* exhibited the highest allelic richness, even surpassing *L.theobromae*, indicating significant adaptability potential. We also observed that the gene diversity of *L.theobromae*, *L.hormozganensis* and *L.iranensis* was higher than previously reported across various regions and host species (Begoude Boyogueno et al. 2012; Al-Sadi et al. 2013; Rêgo et al. 2019). Notably, *L.hormozganensis* showed greater gene diversity than *L.theobromae*, suggesting substantial evolutionary potential in Taiwan. The high genetic diversity amongst all *Lasiodiplodia* species is primarily due to the wide range of host species and geographic regions included in the study. These findings underscore the importance of comprehensive sampling for accurately assessing genetic diversity in *Lasiodiplodia* species.

The reproductive modes of *Lasiodiplodia* species are crucial for understanding their genetic structure and diversity. Although direct observation of sexual stages is rare, molecular techniques provide valuable insights into their reproduction strategies. Our study revealed low but significant values of *I*_A_ and rbarD, along with low clonal fraction values across all *Lasiodiplodia* species. These findings suggest that the *Lasiodiplodia* species which we studied employ a selfing or mixed reproductive system (combining asexual, selfing, occasional sexual and parasexual reproduction) rather than the strictly asexual reproduction suggested by previous studies (McDonald and Linde 2002; Baskarathevan et al. 2012; Bihon et al. 2012a; Bihon et al. 2012b; Rêgo et al. 2019). The observed high genetic diversity and distinct genetic groups indicate a mixed reproductive system, where genetic exchange occurs through hyphal fusion, sexual recombination and parasexual cycles amongst diverse strains. This complex reproductive strategy likely contributes to the adaptability and diversity seen in *Lasiodiplodia* species.

### ﻿Genetic differentiations with low gene flow and a high proportion of shared alleles amongst *Lasiodiplodia* species

The IMa3 analysis revealed generally low gene flow amongst the studied *Lasiodiplodia* species and between genetic groups of *L.theobromae*. This finding contradicts previous hypotheses suggesting extensive gene flow of *L.theobromae* (Mohali et al. 2005; Shah et al. 2010; Boyogueno et al. 2012; Al-Sadi et al. 2013; Mehl et al. 2017). The restricted dispersal may be attributed to the large spore size of *L.theobromae* (21–31 × 13–15.5 μm) (Phillips et al. 2013), which may limit its capacity for long-distance aerial transmission. Field observations support this, showing limited conidia movement in *L.theobromae* vineyards (Úrbez-Torres et al. 2010). Studies of other *Botryosphaeriaceae* species also demonstrate that spores mainly spread through short-distance mechanisms like water splash (Ahimera et al. 2004; Baskarathevan et al. 2012). Although some researchers have proposed wind-mediated dispersal (Mehl et al. 2017; Rêgo et al. 2019), this conclusion stems from studies of related *Botryosphaeriaceae* species rather than *L.theobromae* itself (Swart et al. 1987; Pusey 1989; Amponsah et al. 2009; Úrbez-Torres et al. 2010; Úrbez-Torres 2011; Mehl et al. 2013). Currently, no direct evidence exists for the wind dispersal of *L.theobromae* conidia.

Restricted gene flow alone cannot fully explain the genetic differentiation patterns observed within and amongst *Lasiodiplodia* species. Our analyses revealed substantial admixture amongst *L.theobromae*, *L.hormozganensis* and *L.brasiliensis*, despite limited dispersal. The genetic differentiation patterns showed a stronger correlation with host differences than geographic origin, indicating the influence of natural selection forces. Intriguingly, certai*n Lasiodiplodia* species maintain distinct taxonomic boundaries (Ko et al. 2023) despite sharing a high proportion of alleles. This phenomenon likely results from shared ancestral variation, commonly found in recently diverged species with large effective population sizes (Pamilo and Nei 1988). This hypothesis is supported by our IMa3 analysis, which demonstrated recent divergence and large effective population sizes amongst *Lasiodiplodia* species.

### ﻿Host-associated differentiation of *Lasiodiplodia* species

Our study utilised multiple genetic structure analyses (DAPC, Structure, GENELAND) which consistently revealed distinct cryptic genetic groups within *L.theobromae*. While Structure analysis aligned with DAPC results and GENELAND provided finer-scale details, DAPC was primarily used for defining conservative clusters due to potential overestimation issues with GENELAND (Frantz et al. 2009). The four genetic groups (G1-G4) of *L.theobromae* identified align with host species phylogeny, suggesting host-pathogen co-evolution. Specifically, groups G2 and G3 predominantly comprised isolates from *Myrtaceae* (wax apple, guava), G4 included isolates from *Malvaceae* and *Caricaceae* (cocoa, papaya) and G1 was primarily associated with *Anacardiaceae* (mango).

The four genetic groups' relationships mirror their host phylogenetic patterns (Bdeir et al. 2016; Lin and Chung 2017). This alignment strongly suggests that host species diversity exerts significant disruptive selection pressure, driving genetic differentiation and potentially host specialisation within the pathogen population, reflecting host-pathogen co-evolutionary dynamics. Host variation-induced disruptive selection can drive parasite populations to differentiate, even in sympatry, resulting in new host races or sibling species. This phenomenon is particularly prevalent in predominantly asexual fungi (Kawecki 1998; Giraud et al. 2008).

This observed host-associated differentiation aligns with broader evolutionary hypotheses proposed for the *Botryosphaeriales*. Slippers et al. (2013) proposed that the order *Botryosphaeriales* likely originated in the Cretaceous period and that the majority of its diversification occurred during the Tertiary period. This period coincided with the extensive radiation and expansion of woody angiosperms. They hypothesised that the distribution and evolution of host plants played a significant role in driving the diversification of these fungi (Slippers et al. 2013). De Wet et al. (2008) also suggest that the ancestors of the *Botryosphaeriaceae* initially evolved on angiosperms before later colonising and diversifying on gymnosperms (De Wet et al. 2008). Previous research by De Wet et al. (2008) and Slippers et al. (2013) hypothesised potential host-associated co-evolution within the *Botryosphaeriaceae*, though without direct empirical evidence. Our current study’s findings of distinct genetic groups corresponding to host plant phylogeny and the observed patterns of host-associated differentiation provide additional empirical evidence supporting these earlier hypotheses about host-pathogen co-evolution in *Botryosphaeriaceae*.

The genetic differentiation patterns emphasise natural selection’s role in shaping pathogen diversity. We hypothesise that soft selective sweeps are a key mechanism driving this host-associated adaptation. In these sweeps, genetic diversity persists as selection acts on beneficial alleles that are already present at high frequencies (Garud et al. 2013; Lin et al. 2017). This aligns with our findings of high diversity, increased interspecies differentiation at specific target loci and large, stable effective population sizes across the studied *Lasiodiplodia* species. This mechanism is increasingly recognised as the predominant mode of recent rapid adaptive events in numerous species (Hernandez et al. 2011; Garud et al. 2013; Messer and Petrov 2013; Garud et al. 2015). These conclusions are further supported by previous sequence analysis results (Ko et al. 2023).

## ﻿Conclusions

In conclusion, this study provides a comprehensive investigation into the genetic diversity, structure and evolutionary dynamics of *Lasiodiplodia* species, particularly *L.theobromae*. Transferable microsatellite markers, successfully applied across eight species, proved highly efficient in revealing genetic relationships and fine-scale population structures of these economically significant plant pathogens. Our analyses revealed high genetic diversity across all *Lasiodiplodia* species. Using multiple clustering approaches, we identified cryptic genetic groups within *L.theobromae* and host-associated differentiation across *Lasiodiplodia* species. Notably, genetic structure was influenced more by host specificity than geographic location, suggesting host-driven selection plays a significant role in pathogen evolution. Our findings revealed shared ancestral variation and limited gene flow amongst species, with intraspecific genetic clusters corresponding to host plant phylogeny, lending further support to the hypothesis of host-pathogen co-evolution in *Lasiodiplodia*. These findings contribute to more precise species delimitation and identification methods, enhance taxonomic resolution and provide information for effective management strategies for *Lasiodiplodia* species in agricultural settings. This study establishes a solid foundation for future research on the population genetics and evolutionary biology of *Lasiodiplodia* and related genera within the *Botryosphaeriaceae*.

## ﻿Additional information

### Adherence to national and international regulations

All the fungal strains used in this study have been legally obtained, respecting the Convention on Biological Diversity (Rio Convention).

### Author ORCIDs

Ya-Zhu Ko https://orcid.org/0000-0002-5810-6487

Meng-Shin Shiao https://orcid.org/0000-0001-6655-1700

Yu-Chung Chiang https://orcid.org/0000-0002-0551-9309

### Data availability

The original contributions presented in the study are included in the article/Suppl. material 1 as Raw Data Sheet. Further enquiries can be directed to the corresponding author.

## Supplementary materials

Supplementary material 1 Raw data sheet Data type xlsx Explanation note The original contributions presented in the study. https://binary.pensoft.net/file/1336050This dataset is made available under the Open Database License (http://opendatacommons.org/licenses/odbl/1.0/). The Open Database License (ODbL) is a license agreement intended to allow users to freely share, modify, and use this Dataset while maintaining this same freedom for others, provided that the original source and author(s) are credited.Ya-Zhu Ko, Huei-Chuan Shih, Meng-Shin Shiao, Yu-Chung Chiang

Supplementary material 2 Additional information Data type pdf Explanation note **table S1.** Lists of isolated numbers, locations, hosts and population codes for *Lasiodiplodia*, *Neofusicoccum* and *Botryosphaeria* species. **table S2**. Transferability and allele size range (bp) of microsatellite markers for *Lasiodiplodia* and *Neofusicoccum* species. **table S3**. Estimates of genetic diversity by 16 polymorphic microsatellite loci of *Lasiodiplodia* and *Neofusicoccum* species. *A_r_*: allelic richness, *A_p_*: private allelic richness. **table S4.** List of pairwise genetic distance values (*F*_ST_) (upper diagonal) and the proportion of shared alleles (*D*_PS_) (lower diagonal) amongst *Lasiodiplodia* species, based on microsatellite data. **table S5**. Summary of molecular variance (AMOVA) for microsatellite data of six *Lasiodiplodia* species and *L.theobromae* at distinct hierarchical levels. **table S6**. Mean LnP(K) and *ΔK* for each cluster using Bayesian assignment test in Structure of *Lasiodiplodia* species, based on the sequence and microsatellite data. The bold fonts indicate that the results are adopted and presented in subsequent tables. **table S7**. Estimated the IMa3 Model parameters of *Lasiodiplodia* species, based on the microsatellite data. The phylogenetic topology using the maximum parsimony phylogenetic tree (refers to fig. 23). The species codes are 0: *L.brasiliense*, 1: *L.hormozganensis*, 2: *L.pseudotheobromae*, 3: *L.rubropurpurea*, 4: *L.theobromae*, 5: *L.iraniensis*. The *N*, *M* and *T* are effective population size (*q*), migration rate (*m*) and divergence time (*t*) scaled by the mutation rate (2.80 × 10^-6^–2.50 × 10^-5^ per year). The *N*_0_-*N*_5_ represent the effective population size of species 0-5. The *N*_6_-*N*_10_ correspond to the ancestral effective population sizes on nodes of the tree topology. The *M_i_*_*> j*_ represents the migration rate from species i to species j forwards in time. The *T* means the divergence time on nodes of the tree topology. **table S8**. Estimated the demographic parameters in four genetic groups of *L.theobromae*, based on the microsatellite data by using IMa3. The population topology of four genetic groups was estimated use with the “-j0”. The *N*, *M* and *T* are effective population size (*q*), migration rate (*m*) and divergence time (*t*) scaled by the mutation rate (2.80 × 10^-6^–2.50 × 10^-5^ per year). *N*_0_-*N_3_*: effective population size. *N*_4_-*N*_6_: ancestral effective population sizes on nodes of the population topology. *M_i_*_*> j*_: migration rate from species i to species j forwards in time. *T*: divergence time on nodes of the population topology. The genetic group codes are as follows: 0: G1, 1: G2, 2: G3, 3: G4. **fig. S1**. Principal coordinate analysis (PCoA) graphed in 2-dimensional space, based on SSR-based genetic distance data for (A) six *Lasiodiplodia* species, (B) Six *Lasiodiplodia* species with information on host and infected sites. Coord. 1 and Coord. 2 refer to the first and second principal coordinates, respectively. **fig. S2**. Statistical analyses for determining optimal cluster numbers in *Lasiodiplodia* species using microsatellite data. (A-B) DAPC: Bayesian Information Criterion (BIC) used to infer optimal cluster numbers for (A) six *Lasiodiplodia* species and (B) *L.theobromae*. (C-E) Structure: Delta K values and mean log-likelihood values (LnP(K)) for (C) *Lasiodiplodia* and *Neofusicoccum* species, (D) six *Lasiodiplodia* species and (E) *L.theobromae*. (F-G) GENELAND: Average density of inferred K for (F) *Lasiodiplodia* species and (G) *L.theobromae*. **fig. S3.** Discriminant Analysis of Principal Components and Minimum Spanning Network analyses of *Lasiodiplodia* species, based on microsatellite genotypes. (A) DAPC scatter plot for six *Lasiodiplodia* species and (B) *L.theobromae*. Each dot represents an individual and circles denote different groups identified by DAPC. Inset shows eigenvalues of the analysis. Colours indicate distinct species and genetic groups (G1-G4). Species codes: LBR: *L.brasiliense*, LHO: *L.hormozganensis*, LPSE: *L.pseudotheobromae*, LRU: *L.rubropurpurea*, LTH: *L.theobromae*, LIR: *L.iraniensis*. Numbers in parentheses after species codes represent sample sizes. (C) Minimum Spanning Network analyses of *Lasiodiplodia* species calculated using Bruvo’s distance, based on multilocus genotypes (MLGs). **fig. S4.** Bar plots for the genetic structure of *Lasiodiplodia* and *Neofusicoccum* species, based on microsatellite data generated by Structure. A vertical colour line represents each individual and the same colour indicates that the individual belongs to the same cluster. Black lines separate different species. The species codes are as follows: LBR: *L.brasiliense*, LHO: *L.hormozganensis*, LPSE: *L.pseudotheobromae*, LRU: *L.rubropurpurea*, LTH: *L.theobromae*, LIR: *L.iraniensis*. **fig. S5.** Morphological variability amongst the four genetic groups of *L.theobromae* from different isolations. Culture growing on PDA after four weeks at 25 °C. **fig. S6.** The minimum spanning network calculated via Bruvo’s distance, based on MLGs for microsatellite data of (A) *L.brasiliense*; (B) *L.hormozganensis*; (C) *L.pseudotheobromae*; (D) *L.iraniensis*; and (E) *L.rubropurpurea* using poppr in R. The different colour corresponds to different sample parts (stem and fruit). The red letters represent that the sample is isolated from the stem. **fig. S7.** Multilocus posterior distribution of unscaled demographic parameter estimates for six *Lasiodiplodia* species and four genetic groups of *L.theobromae* based on IMa3 analysis. (A, D) Best-fitting phylogenetic topologies used with -j0 command for *Lasiodiplodia* species and four genotype clusters of *L.theobromae*, respectively; (B, E) Posterior probability distributions for effective population sizes of *Lasiodiplodia* species and four genotype clusters of *L.theobromae*, respectively; (C, F) Posterior probability distributions for migration rates amongst *Lasiodiplodia* species and four genotype clusters of *L.theobromae*, respectively. The species codes are as follows: LBR: *L.brasiliense*, LHO: *L.hormozganensis*, LPSE: *L.pseudotheobromae*, LRU: *L.rubropurpurea*, LTH: *L.theobromae*, LIR: *L.iraniensis*. https://binary.pensoft.net/file/1336051This dataset is made available under the Open Database License (http://opendatacommons.org/licenses/odbl/1.0/). The Open Database License (ODbL) is a license agreement intended to allow users to freely share, modify, and use this Dataset while maintaining this same freedom for others, provided that the original source and author(s) are credited.Ya-Zhu Ko, Huei-Chuan Shih, Meng-Shin Shiao, Yu-Chung Chiang
