# Supplementary material for: ﻿Cryptic host-associated differentiation and diversity: unravelling the evolutionary dynamics of the plant pathogen Lasiodiplodia
Source: IMA Fungus. 2025 May 23;16:e147543. doi: 10.3897/imafungus.16.147543 (PMC12125593; doi:10.3897/imafungus.16.147543)
Supplement: Supplementary material 2 — Additional information [file imafungus-16-e147543-s002.pdf]

Supplementary Table S1. Lists of isolated numbers, locations, hosts, and population codes for *Lasiodiplodia*, *Neofusicoccu*, and *Botryosphaeria* species.

Species	Host	Location	Isolate code	No. of isolates	Cumulative Isolates
Lasiodiplodia theobromae	*Syzygium samarangense* (wax apple)	Linbian Township, Pingtung County	LTH (L)	6	63
		Linbian Township, Pingtung County	LTH (LL)	2	
		Linbian Township, Pingtung County	LTH (LN)	5	
		Changjhih Township, Pingtung County	LTH (KuL)	2	
	Psidium guajava (guava)	Yanchao Dist., Kaohsiung City	LTH (Z)	5	
		Yanchao Dist., Kaohsiung City	LTH (Wa)	3	
		Dajia Dist., Taichung City	LTH (DC)	1	
		Danei Dist., Tainan City	LTH (P)	3	
	Mangifera indica (mango)	Maolin Dist., Kaohsiung City	LTH (ML)	4	
		Nansi Dist., Tainan City	LTH (ZCM)	8	
		Taoyuan Dist., Kaohsiung City	LTH (MM)	3	
		Linluo Township, Pingtung County	LTH (LOM)	1	
	Carica papaya (papaya)	Nansi Dist., Tainan City	LTH (ZCP)	6	
		Kaohsiung City	LTH (MP)	2	
	Annona squamosa (sugar apple)	Taimali Township, Taitung County	LHO (TZS)	2	
		Taimali Township, Taitung County	LHO (TZ)	2	
	Theobroma cacao (cocoa)	Neipu Township, Pingtung County	LHO (PCOCO)	6	
	Cordia dichotoma	Danei Dist., Tainan City	LTH (PP)	1	
	Alpinia	Yanchao Dist., Kaohsiung City	LTH (MT)	1	
Lasiodiplodia brasiliensis	*Syzygium samarangense* (wax apple)	Liouguei Dist., Kaohsiung City	LBR (LK)	8	11
		Neipu Township, Pingtung County	LBR (PLA)	1	
	Mangifera indica (mango)	Taoyuan Dist., Kaohsiung City	LBR (MM)	1	
		Kaohsiung City	LBR (NM)	1	
Lasiodiplodia hormozganensis	*Syzygium samarangense* (wax apple)	Linluo Township, Pingtung County	LHO (LO)	3	18
	Mangifera indica (mango)	Maolin Dist., Kaohsiung City	LHO (ML)	5	
	Psidium guajava (guava)	Dajia Dist., Taichung City	LHO (DC)	2	
		Yanchao Dist., Kaohsiung City	LHO (YBL)	1	
	Annona squamosa (sugar apple)	Taimali Township, Taitung County	LHO (TZS)	5	
	Musa spp. (banana)	Taimali Township, Taitung County	LHO (PbT)	2	
Lasiodiplodia pseudotheobromae	*Syzygium samarangense* (wax apple)	Changjhih Township, Pingtung County	LPSE (KuL)	3	13
	Psidium guajava (guava)	Yanchao Dist., Kaohsiung City	LPSE (Z)	1	
		Tianliao Dist., Kaohsiung City	LPSE (ZLNB)	5	

	Mangifera indicnga (mango)	Maolin Dist., Kaohsiung City	LPSE (ML)	2	
		Taoyuan Dist., Kaohsiung City	LPSE (MM)	1	
		Nansi Dist., Tainan City	LPSE (ZCM)	1	
Lasiodiplodia rubropurpurea	*Syzygium samarangense* (wax apple)	Yanchao Dist., Kaohsiung City	LRU (NYG)	1	26
		Neipu Township, Pingtung County	LRU (PLA)	5	
		Yuanshan Township, Yilan County	LRU (ILE)	2	
	Psidium guajava (guava)	Yanchao Dist., Kaohsiung City	LRU (Z)	2	
		Yanchao Dist., Kaohsiung City	LRU (Wa)	4	
		Yanchao Dist., Kaohsiung City	LRU (Zng)	6	
		Yanchao Dist., Kaohsiung City	LRU (YBL)	2	
		Tianliao Dist., Kaohsiung City	LRU (ZLNB)	2	
	Mangifera indica (mango)	Lucao Township, Chiayi County	LRU (LX)	1	
		Sinying Dist., Tainan City	LRU (NEM)	1	
Lasiodiplodia iranensis	*Mangifera indica* (mango)	Maolin Dist., Kaohsiung City	LIR (ML)	1	14
		Sinying Dist., Tainan City	LIR (NEM)	3	
		Tianliao Dist., Kaohsiung City	LIR (ZLNM)	4	
	Psidium guajava (guava)	Dajia Dist., Taichung City	LIR (DC)	1	
	Annona squamosa (sugar apple)	Taimali Township, Taitung County	LIR (TZ)	2	
	Theobroma cacao (cocoa)	Neipu Township, Pingtung County	LIR (PCOCO)	3	
Neofusicoccum mangiferae	*Syzygium samarangense* (wax apple)	Yanchao Dist., Kaohsiung City	NEOM (NYG)	2	12
		Linbian Township, Pingtung County	NEOM (LN)	1	
	Mangifera indica (mango)	Lucao Township, Chiayi County	NEOM (LX)	3	
		Sinying Dist., Tainan City	NEOM (NEM)	1	
		Neipu Township, Pingtung County	NEOM (PWM)	1	
	Syzygium taiwanicum	Lanyu Township, Taitung County	NEOM (LUBB)	4	
Neofusicoccum parvum	*Syzygium samarangense* (wax apple)	Linbian Township, Pingtung County	NEOP (L)	2	52
		Linbian Township, Pingtung County	NEOP (LL)	2	
		Liouguei Dist., Kaohsiung City	NEOP (LK)	4	
		Meishan Township, Chiayi County	NEOP (Zai)	3	
		Changjhih Township, Pingtung County	NEOP (KuL)	4	
		Linbian Township, Pingtung County	NEOP (LN)	13	
		Xinyi Township, Nantou County	NEOP (NTZE)	22	

Syzygium taiwanicum	Lanyu Township, Taitung County	NEOP (LUBB)	1	
Mangifera indica (mango)	Tianliao Dist., Kaohsiung City	NEOP (ZLNM)	1	
Total				209

Supplementary Table S2. Transferability and allele size range (bp) of microsatellite markers for *Lasiodiplodia* and *Neofusicoccum* species.

Primer	*L. theobromae*	*L. brasiliensis*	*L. hormozganensis*	*L. pseudotheobromae*	*L. rubropurpurea*	*L. iranensis*	*N. parvum*	*N. mangiferae*
LAS1314	318-328	320-326	308-326	302-320	290-328	312-326	248-326	308-334
LAS1516	236-354	332-356	332-350	346-354	232-346	346-360	232-356	234-354
LAS2122	410-440	416-436	410-430	408-418	420-480	408-416	328-446	324-430
LAS2728	446-460	446-450	440-450	446-454	332-538	442-490	420-568	428-552
LAS3536	350-374	356-374	356-382	364-370	360-468	364-382	306-388	320-416
LAS0304	350-376	364-376	360-376	336-368	342-470	330-370	342-394	342-380
LAS1718	250-268	254-268	250-266	252-266	250-256	254-268	205-270	220-268
LAS3334	290-310	288-302	288-310	290-322	288-298	296-322	288-348	270-304
LAS3738	114-144	116-118	114-140	108-144	114-144	116-160	108-150	116-150
LAS2930	180-190	186-188	180-192	176	170-190	176	180-266	170-204
LAS2526	422-432	422-428	422-426	422-428	386-392	422-426	376-524	362-432
LAS3132	412-516	436-456	416-470	430-448	430-458	420-450	346-528	400-456
LAS2324	454-526	454-472	456-482	- (1)	454-532	468-482	400-538	454-542
LAS 01	194-200	196-200	194-198	198-200	196-200	194-200	186-202	196-198
LAS 08	192-198	188-198	186-198	186-200	178-202	192-200	186-242	130-204
LAS 09	136-146	140-146	136-146	140-146	132-146	138-144	138-146	138-146

(1) The symbol indicates that samples could not be successfully amplified.

Supplementary Table S3. Estimates of genetic diversity by 16 polymorphic microsatellite loci of *Lasiodiplodia* and *Neofusicoccum* species. A_r: allelic richness, A_p: private allelic richness.

	L. theobromae		***L. brasiliensis***		***L. hormozganensis***		***L. pseudotheobromae***		***L. rubropurpurea***		***L. iranensis***		***N. parvum***		***N. mangiferae***	
	A_r	A_p	A_r	A_p	A_r	A_p	A_r	A_p	A_r	A_p	A_r	A_p	A_r	A_p	A_r	A_p
LAS1516	4.34	0.00	3.93	0.01	5.21	0.00	3.72	0.25	8.28	1.38	6.28	1.78	6.73	0.84	7.21	1.23
LAS01	2.97	0.00	1.94	0.00	2.68	0.00	2.00	0.00	2.99	0.00	3.78	0.00	4.61	0.68	2.00	0.00
LAS08	3.52	0.00	2.66	0.43	3.88	0.03	2.72	0.02	5.94	1.11	3.80	0.31	5.71	1.26	5.79	2.87
LAS09	4.52	0.07	1.73	0.00	5.14	0.11	3.66	0.00	3.43	0.52	3.97	0.00	4.00	0.00	4.31	0.00
LAS0304	5.98	0.83	2.97	0.05	3.90	0.39	3.72	1.04	7.41	3.34	4.20	2.37	8.17	2.63	4.97	0.70
LAS1314	5.02	0.61	2.94	0.00	5.73	0.74	4.58	0.03	3.00	0.19	4.65	1.00	4.29	2.86	3.00	1.00
LAS1718	5.61	0.19	3.66	0.00	4.60	0.34	5.69	0.02	2.50	0.11	3.37	0.00	7.91	2.47	8.06	2.11
LAS2122	7.23	0.33	3.66	0.00	7.89	0.66	1.86	0.04	6.31	2.13	4.60	1.85	7.67	2.57	7.50	2.60
LAS2324	4.60	0.00	4.87	0.06	5.68	1.51	1.00	0.00	9.18	1.80	3.00	0.00	6.77	2.83	7.00	1.58
LAS2526	2.37	0.00	1.94	0.10	1.98	0.01	1.86	0.04	1.77	1.77	1.98	0.01	5.43	4.18	2.00	1.29
LAS2728	4.15	0.26	2.00	0.00	2.96	0.98	2.97	0.00	11.24	5.52	6.09	2.96	6.13	1.99	6.00	3.38
LAS2930	3.04	0.01	2.73	0.00	5.29	0.90	1.00	0.00	4.98	1.20	1.57	0.00	7.82	5.01	7.11	3.84
LAS3132	9.05	1.38	4.32	0.07	6.27	1.34	5.45	0.04	6.09	0.68	5.47	0.01	7.30	3.20	6.00	0.99
LAS3334	6.24	0.14	3.45	0.03	5.07	1.08	6.49	1.13	3.53	0.00	3.99	0.05	8.00	3.80	8.38	2.65
LAS3536	4.34	0.63	2.93	0.00	5.43	0.07	2.00	0.00	6.59	3.20	4.73	0.02	7.81	4.45	8.00	2.83
LAS3738	6.89	0.65	2.00	0.00	4.39	0.05	7.74	1.06	6.82	0.31	8.41	1.77	7.74	1.49	6.10	1.35
**Mean**	**4.99**	**0.32**	**2.98**	**0.05**	**4.76**	**0.51**	**3.53**	**0.23**	**5.63**	**1.45**	**4.37**	**0.76**	**6.63**	**2.52**	**5.84**	**1.78**

Supplementary Table S4. List of pairwise genetic distance values (F_{ST}) (upper diagonal) and the proportion of shared alleles (D_{PS}) (lower diagonal) among *Lasiodiplodia* species based on microsatellite data.

	L. brasiliense	*L. hormozganensis*	*L. pseudotheobromae*	*L. rubropurpurea*	*L. theobromae*	*L. iraniensis*
L. brasiliense	**-**	0.53	0.75	0.77	0.45	0.75
L. hormozganensis	**0.19**	**-**	0.69	0.68	0.40	0.60
L. pseudotheobromae	**0.48**	**0.32**	**-**	0.73	0.66	0.53
L. rubropurpurea	**0.36**	**0.22**	**0.32**	**-**	0.66	0.76
L. theobromae	**0.14**	**0.07**	**0.30**	**0.22**	**-**	0.64
L. iraniensis	**0.39**	**0.23**	**0.19**	**0.29**	**0.23**	**-**

Supplementary Table S5. Summary of molecular variance (AMOVA) for microsatellite data of six *Lasiodiplodia* species and *L. theobromae* at distinct hierarchical levels.

**Six *Lasiodiplodia* species**					
	Sum of squares	*d.f.*	Variance components	Percentage of variation	Fixation Indices
Among species	222.66	5	0.75	20.59	F_{CT}=0.21*
Among populations within species	421.73	46	1.09	29.84	F_{SC}=0.38*
Among individuals within populations	318.21	94	1.60	43.75	F_{ST}=0.57*
Within individuals	31.00	146	0.21	5.83	
L. theobroma					
	Sum of squares	*d.f.*	Variance components	Percentage of variation	Fixation Indices
Among populations	336.06	18	1.91	36.66	
Among individuals within populations	275.67	44	3.02	57.86	F_{ST}=0.37*
Within individuals	18.00	63	0.29	5.48	

Note: *, $P < 0.05$.

Supplementary Table S6. Mean LnP(K) and *ΔK* for each cluster using Bayesian assignment test in Structure of *Lasiodiplodia* species based on the sequence and microsatellite data. The bold fonts indicate that the results are adopted and presented in subsequent tables.

	**6 *Lasiodiplodia* species**				***L. theobromae***			
	**Sequence**		**Microsatellite**		**Sequence**		**Microsatellite**	
K	Mean LnP(K)	*ΔK*	Mean LnP(K)	*ΔK*	Mean LnP(K)	*ΔK*	Mean LnP(K)	*ΔK*
1	-10302.50	—	-8712.80	—	-517.5	—	-3040.90	—
2	-5483.84	**2941.32**	-7663.34	**2824.00**	-435.58	**142.67**	-2717.60	**249.01**
3	-3475.64	**729.09**	-7126.89	0.63	-454.24	**50.98**	-2504.26	6.66
4	-2309.22	**551.83**	-6613.60	**208.51**	-524.96	2.94	-2239.02	**547.61**
5	-2170.30	0.49	-6355.26	0.69	-419.68	2.99	-2120.72	1.96
6	-2165.08	0.18	-6046.94	3.40	-397.9	0.31	-2006.98	0.71
7	-2117.88	45.88	-5827.92	4.60	-370.82	7.24	-1906.46	0.33
8	-2152.84	34.03	-5665.54	0.81	-399.54	1.45	-1808.34	4.67
9	-2068.06	1.01	-5487.80	4.67	-434.96	1.38	-1732.92	0.51
10	-2232.50	6.08	-5380.00	1.37	-461.12	0.05	-1663.28	0.31
11	-2047.04	1.05	-5206.94	0.59	-487.56	0.16	-1583.82	3.26
12	-2202.02	0.24	-5068.08	0.76	-512.05	—	-1515.45	3.38
13	-2315.56	2.20	-4904.24	4.04			-1456.10	0.90
14	-2355.05	4.34	-4804.70	1.91			-1400.32	1.81
15	-1938.30	—	-4719.80	0.09			-1350.12	3.02
16			-4636.34	0.07			-1311.38	0.69
17			-4550.25	0.20			-1270.70	—
18			-4455.68	2.01				
19			-4402.62	1.28				
20			-4315.54	-				

Supplementary Table S7. Estimated the IMa3 Model parameters of *Lasiodiplodia* species based on the microsatellite data. The phylogenetic topology using the maximum parsimony phylogenetic tree (refers to Figure 23). The species codes are 0: *L. brasiliense*, 1: *L. hormozganensis*, 2: *L. pseudotheobromae*, 3: *L. rubropurpurea*, 4: *L. theobromae*, 5: *L. iraniensis.* The *N*, *M* and *T* are effective population size (*q*), migration rate (*m*) and divergence time (*t*) scaled by the mutation rate (2.80×10^{-6} - 2.50×10^{-5} per year). The N_0-N_5 represent the effective population size of species 0-5. The N_6-N_{10} correspond to the ancestral effective population sizes on nodes of the tree topology. The $M_{i>j}$ represent the migration rate from species i to species j forwards in time. The *T* means the divergence time on nodes of the tree topology.

Parameter	HiPt (2.80×10^{-6} - 2.50×10^{-5})	(2.80×10^{-6}) HPD95Lo-HPD95Hi; (2.50×10^{-5}) HPD95Lo-HPD95Hi
N_0	2232.14 -250.00	357.14 - 4107.14; 40.00 - 460.00
N_1	11160.71 - 1250.00	8214.29 - 17946.43; 920.00 - 2010.00
N_2	22500.00 - 2520.00	15803.57 - 42678.57; 1770.00 - 4780.00
N_3	86250.00 - 9660.00	54821.43 - 154642.86; 6140.00 - 17320.00
N_4	802410.71 - 89870.00	79375.00 - 1100803.57; 8890.000 - 123290.00
N_5	297589.29 - 33330.00	107589.29 - 1408571.43; 12050.00 - 157760.00
N_6	66250.00 - 7420.00	46250.00 - 124732.14; 5180.00 - 13970.00
N_7	50446.43 - 5650.00	37946.43 - 85357.14; 4250.00 - 9560.00
N_8	850982.14 - 95310.00	74464.29 - 1007142.86; 8340.00 - 112800.00
N_9	241696.43 - 27070.00	161607.14 - 374285.71; 18100.00 - 41920.00
N_{10}	2262232.14 - 253370.00	2094732.14 - 2539732.14; 234610.00 - 284450.00
T_0 (LIR & LBR)	178.57 - 20.00	0.00 - 3392.86; 0.00 - 380.00
T_1 (LPSE & LHO)	892.86 - 100.00	535.71 - 5178.57; 60.00 - 580.00
T_2 (LIR & LBR from LTH)	3750.00 - 420.00	1964.29 - 20892.86; 220.00 - 2340.00
T_3 (LIR, LBR & LTH from LPSE & LHO)	6250.00 - 700.00	2678.57 - 28392.86; 300.00 - 3180.00
T_4 (LRU)	8035.71 - 900.00	4107.14 - 42678.57; 460.00 - 4780.00

Supplementary Table S7 (Continue). Estimated the IMa3 Model parameters of *Lasiodiplodia* species based on the microsatellite data. The phylogenetic topology using the maximum parsimony phylogenetic tree (refers to Figure 24). The species codes are 0: *L. brasiliense*, 1: *L. hormozganensis*, 2: *L. pseudotheobromae*, 3: *L. rubropurpurea*, 4: *L. theobromae*, 5: *L. iraniensis.* The *N*, *M* and *T* are effective population size (*q*), migration rate (*m*) and divergence time (*t*) scaled by the mutation rate (2.80×10^{-6} - 2.50×10^{-5} per year). The N_0-N_5 represent the effective population size of species 0-5. The N_6-N_{10} correspond to the ancestral effective population sizes on nodes of the tree topology. The $M_{i>j}$ represent the migration rate from species i to species j forwards in time. The *T* means the divergence time on nodes of the tree topology.

Parameter	HiPt (2.80×10^{-6} - 2.50×10^{-5})	(2.80×10^{-6}) HPD95Lo-HPD95Hi; (2.50×10^{-5}) HPD95Lo-HPD95Hi
$M_{0>1}$	1.54E-04 - 1.38E-03	2.77E-05 - 3.55E-04; 2.48E-04 - 3.17E-03
$M_{1>0}$	5.70E-05 - 5.09E-04	8.37E-06 - 1.21E-04; 7.47E-05 - 1.08E-03
$M_{0>2}$	1.42E-05 - 1.27E-04	9.94E-07 -7.91E-05; 8.88E-06 - 7.06E-04
$M_{2>0}$	0.00E+00 - 0.00E+00	0.00E+00 - 1.79E-05; 0.00E+00 - 1.60E-04
$M_{0>3}$	0.00E+00 - 0.00E+00	0.00E+00 - 2.90E-05; 0.00E+00 - 2.59E-04
$M_{3>0}$	1.88E-05 - 1.68E-04	3.98E-06 - 5.74E-05; 3.55E-05 - 5.12E-04
$M_{0>4}$	2.72E-05 - 2.43E-04	2.93E-06 - 1.23E-04; 2.61E-05 - 1.10E-03
$M_{4>0}$	1.35E-06 - 1.21E-05	1.23E-07 - 1.46E-05; 1.10E-06 - 1.31E-04
$M_{0>5}$	0.00E+00 - 0.00E+00	0.00E+00 - 3.06E-05; 0.00E+00 - 2.73E-04
$M_{5>0}$	0.00E+00 - 0.00E+00	0.00E+00 - 2.07E-05; 0.00E+00 - 1.85E-04
$M_{1>2}$	3.39E-06 - 3.02E-05	5.35E-07 - 1.36E-05; 4.78E-06 - 1.22E-04
$M_{2>1}$	3.51E-06 - 3.14E-05	3.44E-07 - 1.41E-05; 3.08E-06 - 1.26E-04
$M_{1>3}$	3.02E-07 - 2.70E-06	0.00E+00 - 4.78E-06; 0.00E+00 - 4.27E-05
$M_{3>1}$	9.21E-07 - 8.23E-06	5.60E-08 - 6.92E-06; 5.00E-07 - 6.18E-05
$M_{1>4}$	0.00E+00 - 0.00E+00	0.00E+00 - 3.53E-06; 0.00E+00 - 3.15E-05
$M_{4>1}$	0.00E+00 - 0.00E+00	0.00E+00 - 1.30E-06; 0.00E+00 - 1.16E-05
$M_{1>5}$	0.00E+00 - 0.00E+00	0.00E+00 - 9.63E-06; 0.00E+00 - 8.60E-05
$M_{5>1}$	0.00E+00 - 0.00E+00	0.00E+00 - 1.35E-05; 0.00E+00 - 1.21E-04
$M_{2>3}$	1.49E-06 - 1.33E-05	5.88E-08 - 1.25E-05; 5.25E-07 - 1.12E-04
$M_{3>2}$	1.86E-06 - 1.67E-05	2.21E-07 - 7.64E-06; 1.98E-06 - 6.82E-05
$M_{2>4}$	0.00E+00 - 0.00E+00	0.00E+00 - 4.43E-06; 0.00E+00 - 3.95E-05
$M_{4>2}$	0.00E+00 - 0.00E+00	0.00E+00 - 7.90E-07; 0.00E+00 - 7.05E-06
$M_{2>5}$	0.00E+00 - 0.00E+00	0.00E+00 - 1.37E-05; 0.00E+00 - 1.22E-04
$M_{5>2}$	0.00E+00 - 0.00E+00	0.00E+00 - 1.31E-05; 0.00E+00 - 1.17E-04
$M_{3>4}$	1.65E-06 - 1.48E-05	5.26E-07 - 5.60E-06; 4.70E-06 - 5.00E-05
$M_{4>3}$	3.43E-06 - 3.06E-05	3.95E-07 - 6.08E-06; 3.53E-06 - 5.43E-05
$M_{3>5}$	0.00E+00 - 0.00E+00	0.00E+00 - 7.05E-06; 0.00E+00 - 6.29E-05
$M_{5>3}$	0.00E+00 - 0.00E+00	0.00E+00 - 1.20E-05; 0.00E+00 - 1.07E-04
$M_{4>5}$	0.00E+00 - 0.00E+00	0.00E+00 - 4.62E-06; 0.00E+00 - 4.13E-05
$M_{5>4}$	0.00E+00 - 0.00E+00	0.00E+00 - 1.38E-05; 0.00E+00 - 1.23E-04

Supplementary Table S8. Estimated the demographic parameters in four genetic groups of *L. theobromae* based on the microsatellite data by using IMa3. The population topology of four genetic groups was estimated use with the"-j0". The *N*, *M* and *T* are effective population size (*q*), migration rate (*m*) and divergence time (*t*) scaled by the mutation rate (2.80×10^{-6} - 2.50×10^{-5} per year). N_0-N_3: effective population size. N_4-N_6: ancestral effective population sizes on nodes of the population topology. $M_{i>j}$: migration rate from species i to species j forwards in time. *T*: divergence time on nodes of the population topology. The genetic group codes are as follows: 0: G1, 1: G2, 2: G3, 3: G4.

Four genotypes of *L. theobromae* - population Tree: (3,(1,(0,2)4)5)6		
Parameter	HiPt (2.80×10^{-6} - 2.50×10^{-5})	(2.80×10^{-6}) HPD95Lo-HPD95Hi; (2.50×10^{-5}) HPD95Lo-HPD95Hi
N_0	5357.14 - 600.00	0.00 - 91071.43; 0.00 - 10200.00
N_1	5357.14 - 600.00	0.00 - 91071.43; 0.00 - 10200.00
N_2	37500.00 - 4200.00	16071.43 - 219642.86; 1800.00 - 24600.00
N_3	37500.00 - 4200.00	26785.71 - 166071.43; 3000.00 - 18600.00
N_4	16071.43 - 1800.00	16071.43 - 123214.29; 1800.00 - 13800.00
N_5	91071.43 - 10200.00	48214.29 - 219642.86; 5400.00 - 24600.00
N_6	1419642.86 - 159000.00	1173214.29 - 1783928.57; 131400.00 - 9800.00
T_0	178.57 - 20.00	0.00 - 0.00; 0.00 - 380.00
T_1	892.86 - 100.00	535.71 - 5178.57; 60.00 - 580.00
T_2	6964.29 - 780.00	3750.00 - 15892.86; 420.00 - 1780.00
$M_{0>1}$	1.43E-05 - 1.28E-04	1.40E-06 - 6.75E-05; 1.25E-05 - 6.03E-04
$M_{1>0}$	3.00E-05 - 2.68E-04	5.32E-06 - 1.85E-04; 4.75E-05 - 1.65E-03
$M_{0>2}$	1.60E-05 - 1.43E-04	2.52E-06 - 8.93E-05 ; 2.25E-05 - 7.98E-04
$M_{2>0}$	2.52E-06 - 2.25E-05	0.00E+00 - 3.11E-05; 0.00E+00 - 2.78E-04
$M_{0>3}$	2.80E-07 - 2.50E-06	0.00E+00 - 3.84E-05; 0.00E+00 - 3.43E-04
$M_{3>0}$	2.80E-07 - 2.50E-06	0.00E+00 - 3.22E-05; 0.00E+00 - 2.88E-04
$M_{1>2}$	2.80E-07 - 2.50E-06	0.00E+00 - 5.29E-05; 0.00E+00 - 4.73E-04
$M_{2>1}$	1.60E-05 - 1.43E-04	3.64E-06 - 1.16E-04; 3.25E-05 - 1.03E-03
$M_{1>3}$	7.00E-06 - 6.25E-05	8.40E-07 - 3.50E-05; 7.50E-06 - 3.13E-04
$M_{3>1}$	8.40E-07 - 7.50E-06	0.00E+00 - 8.68E-06; 0.00E+00 - 7.75E-05
$M_{2>3}$	5.88E-06 - 5.25E-05	0.00E+00 - 5.29E-05; 0.00E+00 - 4.73E-04
$M_{3>2}$	2.80E-07 - 2.50E-06	0.00E+00 - 3.05E-05; 0.00E+00 - 2.73E-04

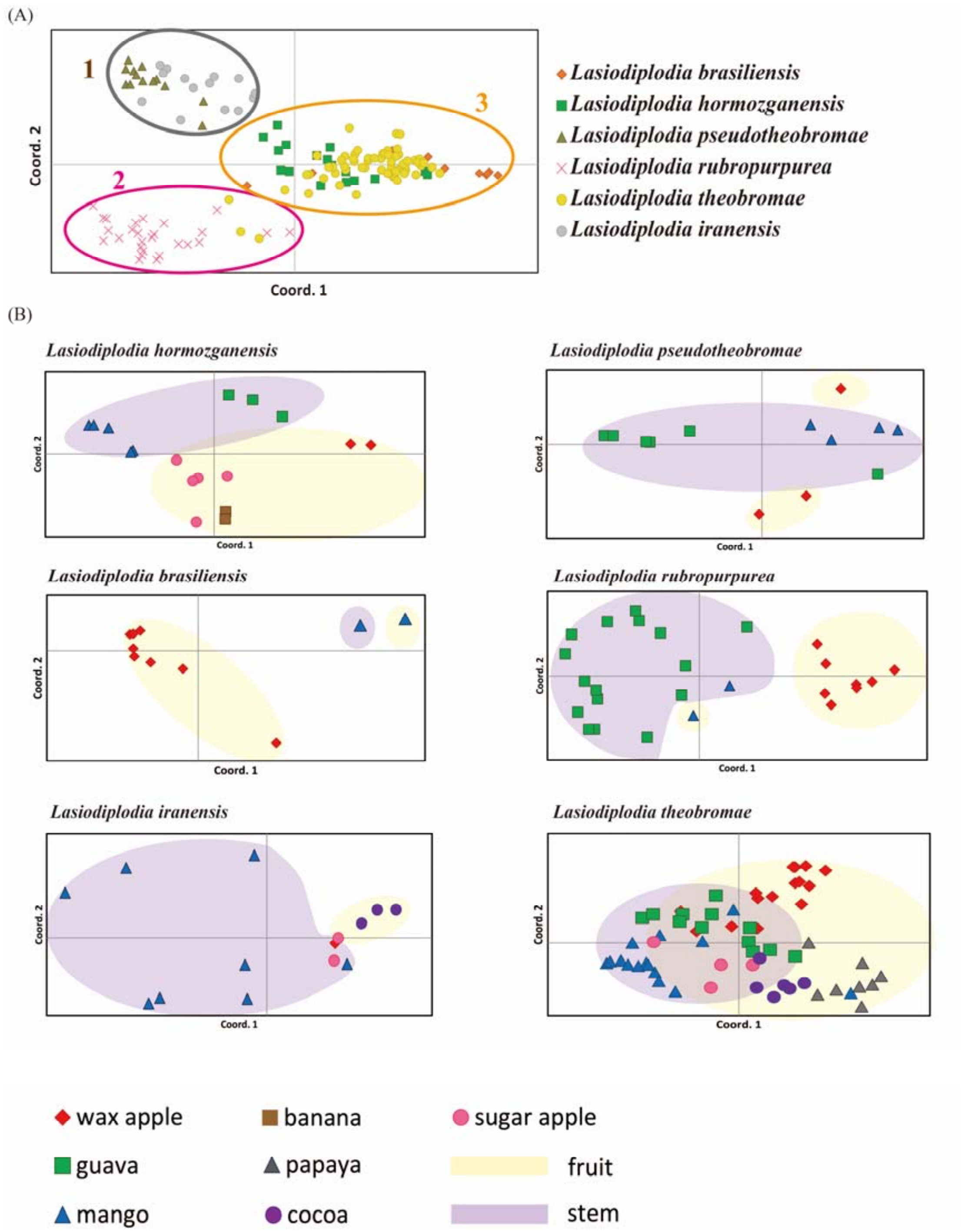

Supplementary Fig. S1. Principal coordinate analysis (PCoA) graphed in 2-dimensional space based on SSR-based genetic distance data for (A) six *Lasiodiplodia* species, (B) Six *Lasiodiplodia* species with information on host and infected sites. Coord. 1 and Coord. 2 refer to the first and second principal coordinates, respectively.

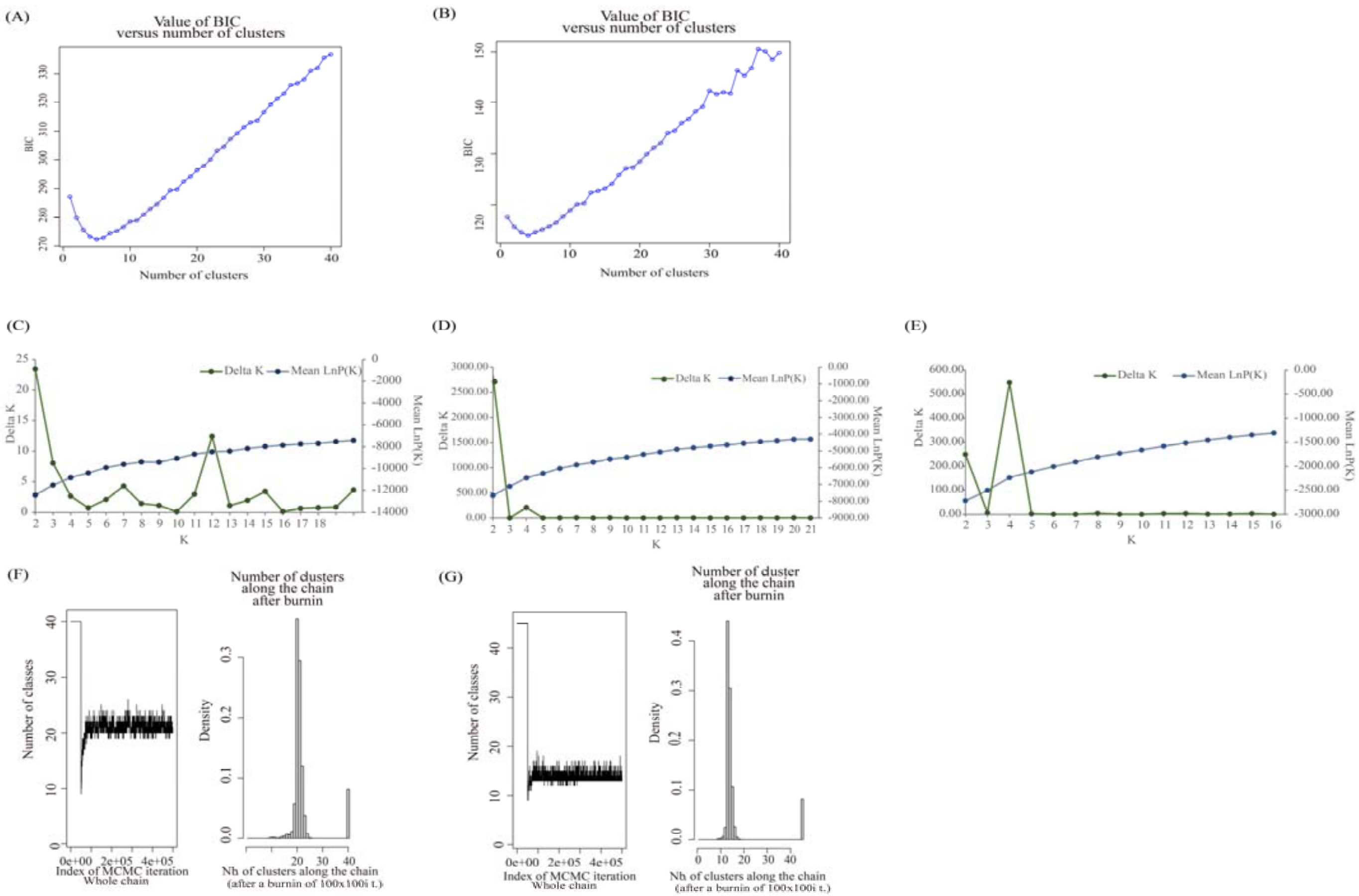

Supplementary Fig. S2. Statistical analyses for determining optimal cluster numbers in *Lasiodiplodia* species using microsatellite data. (A-B) DAPC: Bayesian Information Criterion (BIC) used to infer optimal cluster numbers for (A) six *Lasiodiplodia* species and (B) *L. theobromae*. (C-E) Structure: Delta K values and mean log-likelihood values (LnP(K)) for (C) *Lasiodiplodia* and *Neofusicoccum* species, (D) six *Lasiodiplodia* species, and (E) *L. theobromae*. (F-G) GENELAND: Average density of inferred K for (F) *Lasiodiplodia* species and (G) *L. theobromae*.

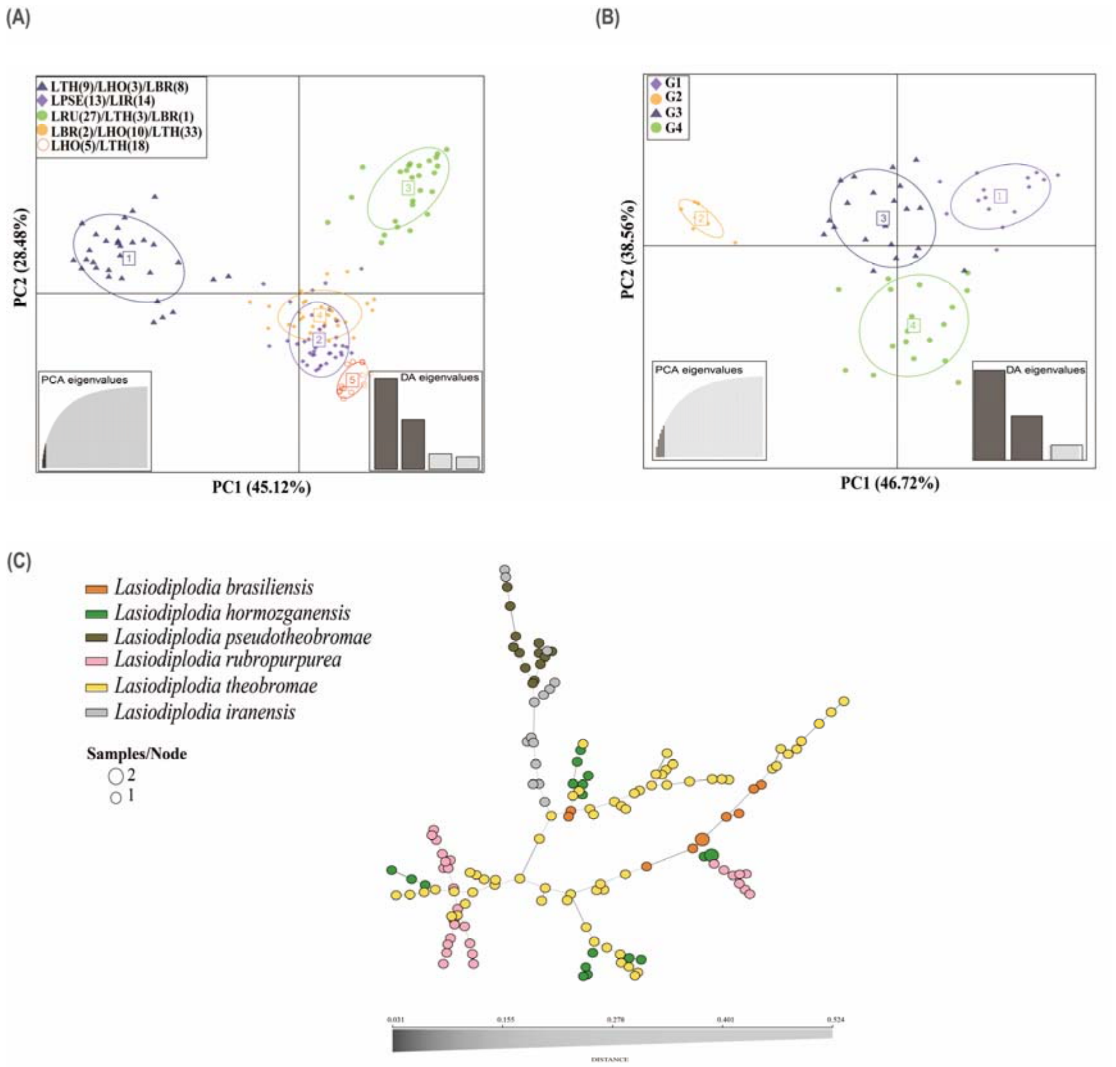

Supplementary Fig. S3. Discriminant Analysis of Principal Components and Minimum Spanning Network analyses of *Lasiodiplodia* species based on microsatellite genotypes. (A) DAPC scatter plot for six *Lasiodiplodia* species and (B) *L. theobromae*. Each dot represents an individual, and circles denote different groups identified by DAPC. Inset shows eigenvalues of the analysis. Colors indicate distinct species and genetic groups (G1-G4). Species codes: LBR: *L. brasiliense*, LHO: *L. hormozganensis*, LPSE: *L. pseudotheobromae*, LRU: *L. rubropurpurea*, LTH: *L. theobromae*, LIR: *L. iraniensis*. Numbers in parentheses after species codes represent sample sizes. (C) Minimum Spanning Network analyses of *Lasiodiplodia* species calculated using Bruvo's distance based on multilocus genotypes (MLGs).

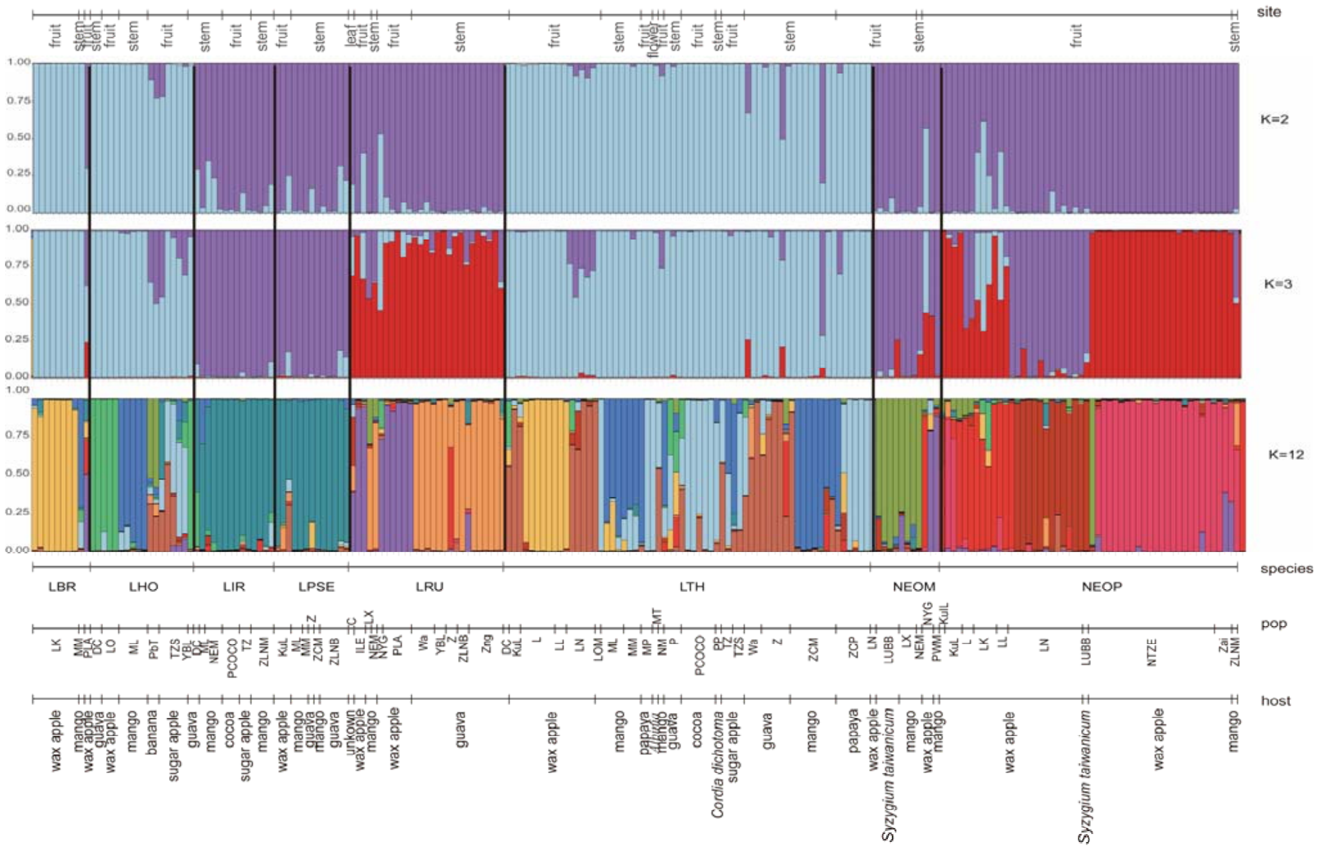

Supplementary Fig. S4. Bar plots for the genetic structure of *Lasiodiplodia* and *Neofusicoccum* species based on microsatellite data generated by Structure. A vertical colour line represents each individual, and the same colour indicates that the individual belongs to the same cluster. Black lines separate different species. The species codes are as follows: LBR: *L. brasiliense*, LHO: *L. hormozganensis*, LPSE: *L. pseudotheobromae*, LRU: *L. rubropurpurea*, LTH: *L. theobromae*, LIR: *L. iraniensis*.

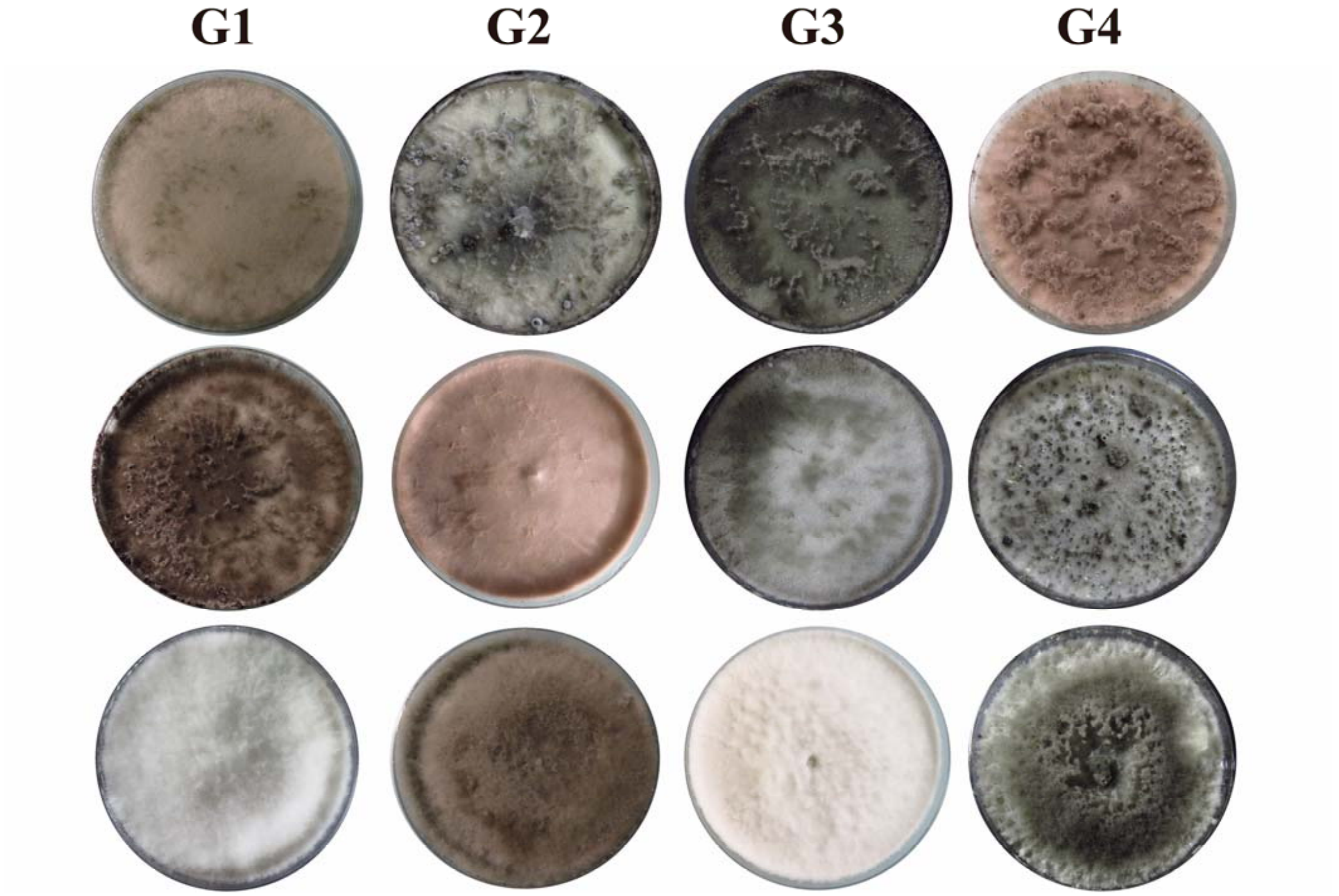

Supplementary Fig. S5. Morphological variability among the four genetic groups of *L. theobromae* from different isolations. Culture growing on PDA after four weeks at 25 °C.

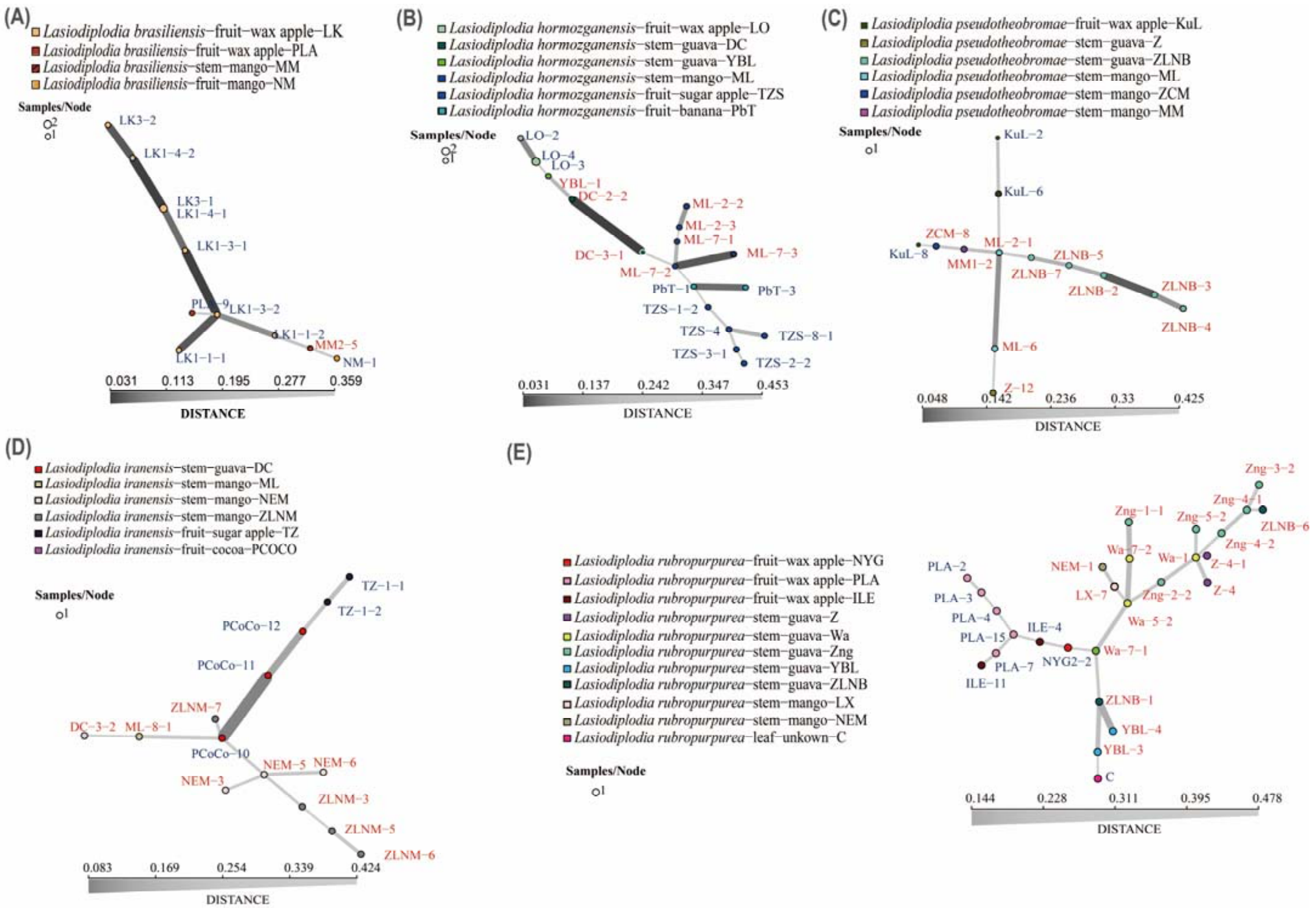

Supplementary Fig. S6. The minimum spanning network calculated via Bruvo's distance based on MLGs for microsatellite data of (A) *L. brasiliense* (B) *L. hormozganensis* (C) *L. pseudotheobroma* (D) *L. iraniensis* (E) *L. rubropurpurea* using poppr in R. The different colour corresponds to different sample parts (stem and fruit). The red letters represent that the sample is isolated from the stem.

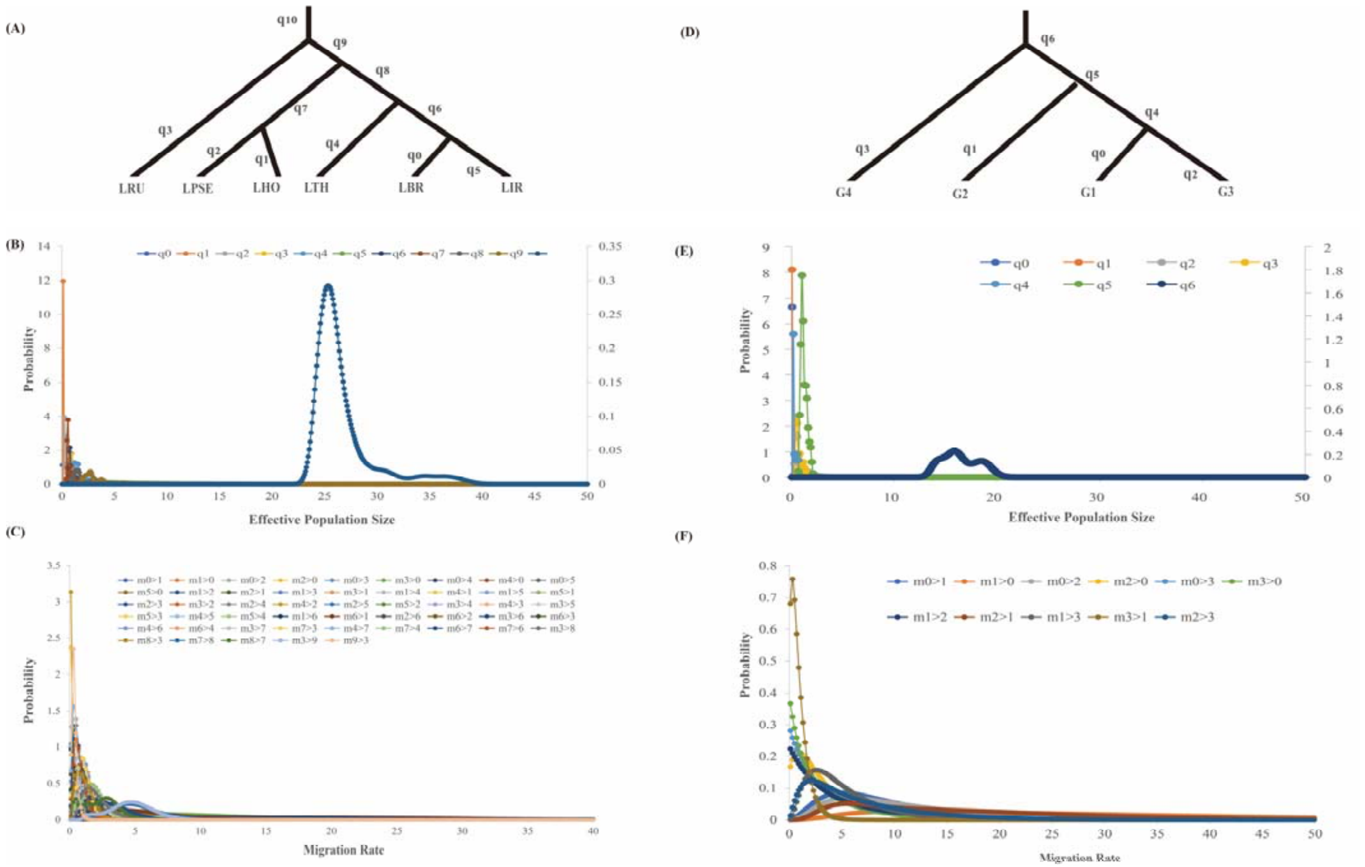

Supplementary Fig. S7. Multilocus posterior distribution of unscaled demographic parameter estimates for six *Lasiodiplodia* species and four genetic groups of *L. theobromae* based on IMa3 analysis. (A, D) Best-fitting phylogenetic topologies used with -j0 command for *Lasiodiplodia* species and four genotype clusters of *L. theobromae*, respectively. (B, E) Posterior probability distributions for effective population sizes of *Lasiodiplodia* species and four genotype clusters of *L. theobromae*, respectively. (C, F) Posterior probability distributions for migration rates among *Lasiodiplodia* species and four genotype clusters of *L. theobromae*, respectively. The species codes are as follows: LBR: *L. brasiliense*, LHO: *L. hormozganensis*, LPSE: *L. pseudotheobromae*, LRU: *L. rubropurpurea*, LTH: *L. theobromae*, LIR: *L. iraniensis*.
